# Supplementary material for: Automatic detection of 39 fundus diseases and conditions in retinal photographs using deep neural networks
Source: Nat Commun. 2021 Aug 10;12:4828. doi: 10.1038/s41467-021-25138-w (PMC8355164; doi:10.1038/s41467-021-25138-w)
Supplement: Supplementary file 1 — Supplementary Information [file 41467_2021_25138_MOESM1_ESM.pdf]

Supplementary Information：

Automatic detection of 39 fundus diseases and conditions in retinal photographs using deep neural networks

Ling-Ping Cen[1 #], Jie Ji[2,3,4 #], Jian-Wei Lin[1], Si-Tong Ju[1], Hong-Jie Lin[1], Tai-Ping Li[1], Yun Wang[1], Jian-Feng Yang[1], Yu-Fen Liu[1], Shaoying Tan[1], Li Tan[1], Dongjie Li[1], Yifan Wang[1], Dezhi Zheng[1], Yongqun Xiong[1], Hanfu Wu[1], Jingjing Jiang[1], Zhenggen Wu[1], Dingguo Huang[1], Tingkun Shi[1], Binyao Chen[1], Jianling Yang[1], Xiaoling Zhang[1], Li Luo[1], Chukai Huang[1], Guihua Zhang[1], Yuqiang Huang[1], Tsz Kin Ng[1,3,5], , Haoyu Chen[1], Weiqi Chen[1], Chi Pui Pang[1,5], Mingzhi Zhang[1]*

[1]Joint Shantou International Eye Centre of Shantou University and The Chinese University of Hong Kong, Shantou, Guangdong, China
[2] Network & Information Centre, Shantou University, Shantou, Guangdong, China
[3] Shantou University Medical College, Shantou, Guangdong, China
[4] XuanShi Med Tech (Shanghai) Company Limited, Shanghai, China
[5] Department of Ophthalmology and Visual Sciences, The Chinese University of Hong Kong, Hong Kong

Supplementary Methods

Algorithm development and the DLP deployment

Multi-label classification and multi-class classification

A two-level hierarchical classification system was used to classify fundus images. Multi-label[1] classification was used for bigclass and multi-class classification for subclasses. As for the multi-label classification, sigmoid was used as the last layer's activation function, a neural network outputted multiple probability values (one for every class), and weighted binary cross-entropy was used as the loss function[2]. Compared with multiple independent classifiers[2], this setting can be viewed as a multi task learning with hard parameter sharing, which can reduce the risk of overfitting[3]. An explicit Non-referable class was included in the bigclasses. T-Criterion rule[4] was used to deal with the all-negative-score cases based on the Close World Assumption, i.e., all examples belong to at least one class. As for the multi-class classification, softmax was used as the last layer's activation function and weighted categorical cross-entropy was used as the loss function.

The bigclass dataset was extremely imbalanced (Supplementary Fig. 5), partly because some labels were more frequent than the others (inter class), and partly due to sparse labels. During training, dynamic data resampling[5] and weighted binary cross-entropy loss function were used to tackle the issue of imbalance of classes. The imbalance ratio values of subclass datasets were not as high as that of bigclass dataset, so only dynamic data resampling was used to tackle imbalance of subclasses.

Fundus image quality assessment

Image quality was calculated by a traditional feature engineering and machine learning method adapted from H. Davis' method.[6] Briefly, 7 areas were extracted from each fundus image, and then 17 features were extracted from each color space (RGB or CIELAB) in each area. The ultimate image quality value was a linear weighted average of these feature variables.

Image preprocessing

The algorithm of image preprocessing was in three steps. Firstly, black background areas were cropped. Secondly, a Hough Circle transformation was used to detect the circle of the retina. If the circle was not correctly found due to bad image quality, the center of the image was assumed to be the center of the circle, and the radius of the circle was obtained based on the pixel distribution of the middle horizontal line[7]. The retina area was then extracted based on the retina circle. Thirdly, to avoid deleting meaningful areas during image augmentation, some black areas (6% of the length of the shortest side of the image) were added to the four borders of a fundus image. Code for image preprocessing has been uploaded to Github (https://github.com/linchundan88/Fundus-image-preprocessing).

Optic disc segmentation dataset

The optic disc segmentation dataset were constructed using both public dataset including REFUGE[8], IDRiD[9], DRIONS-DB[10] and private dataset. The dataset contained a total of 1792 samples and the private dataset contained 700 samples. Images in the private dataset were randomly selected from the classification dataset and excluded images belong to bigclass 10. The private dataset was randomly split into four subsets of the same sample size. Four research assistants who had been trained by doctors participated in the labeling process. Each individual independently annotated a subset. Labelme software was used to label these images.

Convolutional neural networks

Four CNN groups and a Mask R-CNN[11] were used (Supplementary Table 4). Group A was used in all bigclass and subclasses classification with the following exceptions. Group B was only used to divide bigclass 0 into "Normal" and "DR1". Group C accompanied with Mask R-CNN were only used to classify bigclass 10 into possible glaucoma and optic atrophy. Group D was used in some very easy tasks such as distinguishing left and right eyes. To ensure the diversity of models, CNNs with different architecture were used in every CNN group. Ensemble learning was best suited for models were high accuracy and different[12].

CNNs in the group A and group D were "standard" models , the former included Inception-V3[13], Xception[14] and InceptionResNet-V2[15], and the latter MobileNetV2[16] and MnasNet[17]. CNNs in Groups B and C were custom designed models. Their architecture were based on ResNet[18] and ResNeXt[19]., To detect tiny microaneurysms in Group B, input image resolution was enlarged to 448x448, which was doubled as that used in standard VGG and ResNet[18]. To

match the double sized input shape, an addition Conv block was added. The filters of the first convolution layer were reduced from 64 to 32, and the kernel size of the first convolutional layer changed from 7x7 to 5x5. In the custom designed ResNext model cardinality=16 was used instead of cardinality=32. Every Conv block contained a number of residual units (or grouped residual units). Inside every residual units, pre-activation[20] and bottleneck structures were used. The structures of the CNNs in the group B were shown in Supplementary Fig. 3a.

The subclass classification of bigclass10 was considered as a fine-grained classification and was implemented by a small pipeline. An image was firstly pre-processed to be 384x384 pixels, and then a Mask R-CNN was used to detect and segment the optic disc. The optic disc detection task was considered as an instance segmentation problem instead of a localization or object detection problem. This was because the confidence value outputted by Mask-RCNN was important and the mask images of optic disc were easy to obtain. After cropping the optic disc area to 112x112 pixels, a custom designed Resnet and ResNeXt were used to do the final classification task. Compared with the standard Resnet and ResNext, there were some modifications: a Conv block was removed to match half sized input shape and the kernel size of the first convolutional layer was reduced from 7x7 to 5x5. The structures of the CNNs in Group C were shown in Supplementary Fig. 3b. Instance segmentation was used instead of object detection and semantic segmentation because there was a large number of pixel-level annotated data samples and the confidence value of the detected optic disc was very important.

Real time data augmentation

Real time data augmentation was used during both training and inference. In general, image augmentation during training was much more used than during test time. However, test time image augmentation has been used in ImageNet[21] (multi-crop) and Kaggle Data Science Bowl 2017 competitions[22]. It improved not only the accuracy but also robustness to small image perturbation[23]. Compared with before-hand image augmentation, real time image augmentation was flexible and simplified the whole training process. During training, images were randomly rotated (range: [-15◦, 15◦]), translated (range:[-10 %, 10 %]), scaled (range: [95%,105%]) [24], horizontally and vertically flipped, and image contrast were modified (multiplicative factor range:[90%,110%]). Whereas during inference, for an image, two other images were generated on the fly using pre-defined transformations. One image was generated by moving (dx=6px, dy=6px) and horizontal flipping, and the other image was generated using moving (*dx*=-6px, *dy*=-6px) and vertical flipping. Training time image augmentation was implemented using the imgaug[25] library and Keras[26] Sequence(better than python generator in multi-process environment), and test time image augmentation was implemented by custom designed OpenCV codes and Keras Sequence.

Dynamic data resampling

During the training process, dynamic data resampling was used to resolve the problem of imbalanced classes [5]. Compared with traditional under-sampling, it can make full use of training data because in every epoch it generates a different training dataset. Compared with traditional over-sampling, which simply duplicate minority class samples, it can avoid overfitting. Because dynamic resampling and real time augmentation were used together, different images were generated on the fly using real time augmentation for a minority class image. Data resampling methods were widely used in single label setting, however it could not be directly used in multi-label setting. When calculating the sampling probability of an image with multiple labels, the labels was converted to a single label, which was the class with the smallest data samples among the image's multiple labels. It should be noted that this conversion was only used in the sampling process, when generating the training dataset, the original labels wound be used. This method worked well because labels of bigclass dataset were very sparse (Label cardinality = 1.098). The dynamic data resampling algorithm was shown in Algorithm 1.

```
Let S be the original training set.
Let epoch_num  be the epoch of training.
num_classes ←30   # set  num_classes  to be the number of classes of S(in this case 30).
num_samples ⟵  len(S)   # set  num_samples  to be the number of samples in S

S' = copy.deepcopy(S)   #clone a new set object  S'  using  S.
FOR each  sample1  in  S'
          IF len(sample1[1])>1   #sample1 contains data and labels (x, y), determine y in sample1 has multiple labels or not.
                select the label with the minimum class samples, and then replace the original labels by the selected label.
          ENDIF
ENDFOR

class_samples← [ ]
FOR  i=0  to  num_classes-1
     set  class_samples[i]  to be the sample size of class i in S'
ENDFOR

FOR  epoch_current=0  to  epoch_num–1
     #using weight_power to determine the sampling probability of each class. This  parameter can change during training.
     set the weight_power  parameter by reading configuration file or dynamically change it according to the predefined rule.
     p ← [ ]    #p is a list of class resampling probabilities
     FOR  i=0  to  num_classes–1
          p[i]  ⟵ (max(class_samples)**weight_power)/(class_samples[i] **weight_power)
     ENDFOR

     Ŝ ⟵ new set()    #generating a new set Ŝ  , and it will be the training set of the current epoch.
     j ←0
     k ←0
     WHILE True
          randomly select S[j]  using probability p[class1]
          IF  S[k] is selected
               add  S[k]  to  Ŝ
               k ← k + 1
               IF k == num_samples   #The sampling process in this epoch has completed.
                    break
               ENDIF
          ENDIF

          IF j == num_samples   #set the sampling index to be zero, a sample can be sampled multiple times.
               j ← 0
          ENDIF
          j ← j + 1
     END

     yield Ŝ   #return the training dataset of this epoch from to the training process.
ENDFOR
```

Algorithm 1: Dynamic data resampling

In this study, the weight_power parameter was set to 0.65 for bigclass classification. For subclass classifications, dynamic data resampling ratio parameters were set case by case. There parameters were kept stable during training.

Loss function for Multi-label classification

A custom designed function which could be viewed as the weighted binary cross entropy loss was used in bigclass classification. The loss function was formally defined as follows: A single sample in the training set was denoted by ($\boldsymbol{x}$, $\boldsymbol{y}$). The No. *C* output of a neural network f($\boldsymbol{x}$) was denoted by $\boldsymbol{p}_c$, and the No. *C* label of ground truth was denoted by $\mathbf{y}_c$. Because of label smoothing[27], the element $\boldsymbol{y}_c$ was not always be 0 or 1. The loss of ($\boldsymbol{x}$,$\boldsymbol{y}$) was denoted by L($\boldsymbol{x}$, $\boldsymbol{y}$). False negative and false positive weights in the cost matrix of class c were denoted by C_{FNc} and C_{FPc} respectively.

$$\mathrm{L}(\boldsymbol{x}, \boldsymbol{y}) = \sum_{c=1}^{30}(-C_{\mathrm{FNc}}\boldsymbol{y}_c \log(\boldsymbol{p}_c) - C_{\mathrm{FPc}}(1-\boldsymbol{y}_c)(1-\boldsymbol{p}_c))$$

For simplicity, C_{FPc} was set to 1 for all classes, so only C_{FN} needs to be set. C_{FN} which contained 30 numbers were set automatically based on two hyper-parameters: positive_weight_ratio and weight_power.

The algorithm of computing C_{FN}.

class_weight$_c$ =(max_class_samples ** weight_power) / (positive_samples$_c$ ** weight_power)

positive_weight_ratio = 2.4

C_{FNc} = positive_weight_ratio * class_weight$_c$

The class_weight parameters were used to tackle the inter-class imbalance that some labels were more frequent than the others. The class weight for class No. *C* in the loss function was denoted by class_weight$_c$. max_class_samples was the positive sample number of the class with the most positive samples. The positive sample number of the class No. *C*. was denoted by positive_samples$_c$. The weight_power hyper-parameter was set to 0.11. Hyper-parameter positive_weight_ratio was used to tackle the class imbalanced between negative and positive brought by labels sparsity and binary relevance conversion, and it was empirically set to 2.4.

Transfer learning and optimization methods

Transfer learning[28,29] was applied for training standard models in the group A and D. All custom-defined models in the CNN group B and C were trained from scratch (Supplementary Table 4).

During training bigclass models in the group A and all models in the group D, weights were initialized using pre-trained ImageNet models (except for weights of the last fully connected layer), and then all layers were fine-tuned. Bigclass models were trained prior to training subclass models, the subclass models in the CNN group A were transferred from big class models using the method mentioned previously. The domains of subclass classification were subsets of that of the bigclass classification. The tasks of subclass classification and bigclass classification were related. The more similar the data distribution between source domain and target domain and the more related the source task and the target task, the better was the transferring effect.

Adam[30] with lookhead[31](k=5, alpha=0.5) was used as the optimizer, and a custom learning rate scheduler was used to adjust learning rate dynamically. Label smoothing[27] (ε=0.1) was used to calibrate predicted probabilities[32].

Prediction Process

Model ensemble

For an image, after being preprocessed, test time image augmentation and model ensemble were used to generate the final predicted probabilities (Supplementary Fig. 4). The mathematical formula:

$$\text{probs} = \frac{\sum_{i=1}^{n} \sum_{j=1}^{m}(W_i \times \boldsymbol{P}_{ij})}{(\sum_{i=1}^{n} W_i)\times m}$$

The number of CNN models involved was denoted by n and the times of test time image augmentation was denoted by m. W_i was the weight of the model No. *i*. For simplicity, instead of being learned by a meta-learner[33], W_i was set as the square of the validation accuracy of model *i*. $\boldsymbol{P}_{ij}$ was the predicted probability of model *i* for image augmentation *j*. Both parameter *n* and *m* were set to 3. Setting *n* and *m* to be greater than 3 will not generate apparent performance improvement, however it will results in consuming more computing power and long response time. The final predicted probabilities array was denoted by probs.

Generating labels from predicted probabilities

The algorithm of generating bigclass labels was shown in Algorithm 2.

```
Let probs be the predicted probabilities (after model ensembling)

# 0.5 was used as the threshold of positive and negative classes
list_thresholds =np.array([0.5 for _ in range(30)])
#get predicted big classes including the non-referable class.
list_classes = probs > list_thresholds

#get predicted disease classes (non-referable class was excluded).
list_disease_classes = list_classes[1:]

'''if a sample was predicted as negative for all disease classes, select the class(including the non-referable class) with the maximum probability'''
IF len(list_disease_classes==True)==0
    list_disease_classes.append(probs.argmax(axis=-1))
ENDIF

return list_disease_classes
```

Algorithm 2: How to generate bigclass labels

Subclass classification(multi-class) algorithm:

pred_class = probs.argmax(axis=-1)

The predicted class was denoted by pred_class. As for bigclass classification (multi-labels), for simplicity, threshold-moving was not adopted and 0.5 was used as the threshold for all classes. Moreover, abovementioned T-Criterion rule[4] was implemented in the multi-label classification algorithm.

Visualizing and explaining CNNs

The explainability of neural networks was very important, unfortunately all current explanation methods were fragile[34]. A modified Class Activation Maps (CAMs)[35] and the DeepShap[36](DeepExplainer), which can complement each other, were simultaneously used to generate heat-maps (Fig. 1). These heat-maps were used to explain decisions made by neural networks. Class Activation Maps (CAMs)[35] were class discriminative and faithful to predicted values，but with low resolution. DeepExplainer was a combination of Deeplift[37] and Shapley value. It could generate fine-grained heat-maps and was more efficient. Accordingly it could generate more reliable results than other approximation methods such as Layer-wise relevance propagation (LRP) and Integrated Gradients[38]. The differences between the original CAMs and our modified CAMs were only two RELU functions.

The activation of unit k in the last convolutional layer at spatial location (*x*, *y*) was denoted by $f_k(x,y)$, and the weight corresponding to class *c* for unit *k* was denoted by w_k^c . The mathematical formula of CAM was changed from $\mathrm{M_c(X,Y)} = \sum_k w_k^c \ f_k(x,y)$ to $\mathrm{M_c(X,Y)} = \mathrm{ReLU}\left(\sum_k \mathrm{ReLU}(w_k^c)\, f_k(x,y)\right)$. The intuition was the same as Guided Backpropagation and Grad-CAM++[39], i.e., only the positive gradients (or positive weights of the last fully connected layer) were taken into consideration. According to our experimental results, performance of the modified CAMs was obviously better than the original CAMs.

The DLP deployment

After being fully trained and validated, all the CNN models were deployed for production. The simplified architecture of the production platform was shown in Supplementary Fig. 1. A custom designed computer-aided diagnosis service (CADS) was developed instead of using standard Tensorflow Serving because generating heat-maps needs low-level controls on models. Trained CNN models were automatically loaded during the startup of theCADS and provided services through the xmlrpc server. Both web app and web service implemented an xmlrpc client that communicate with CADS (Supplementary Fig. 7). Web service is a restful service, doctor station communicates with it using http protocol. Python3 build-in xmlrpc was used to develop the RPC server, and Django framework was applied to

develop web application and web service.

Development and deployment environment

Development environment:

Hardware: Intel i7-770K, 64GB Memory, four GPUs (1 Nvidia GeForce GTX 1080 and 3 GTX 1080ti and 1 RTX 2080 Ti).

Software: Ubuntu 16.04, CUDA 9.2, cuDNN7.2.1, Tensorflow_gpu version 1.12, Keras2.2.4, MySQL Server (5.7.23), Anaconda5.2.0.

Deployment environment:

Hardware: Intel E5-2620 V4 * 2, 64GB Memory, 1 GeForce GTX 1080 Ti only used for Deep Shap)

Software: Ubuntu 16.04, Intel MKL, intel optimization for Tensorflow_cpu 1.12, CUDA 9.2, cuDNN7.2.1, Tensorflow gpu version 1.12, MySQL Server (5.7.23), Anaconda5.2.0.

Programming languages frameworks and libraries:

Python3.5, C++, OpenCV, TensorFlow[40], Keras, NumPY, Sklearn, SciPY, Matplotlib, Django, Pandas, Imgaug, Shap.

Supplementary Figures

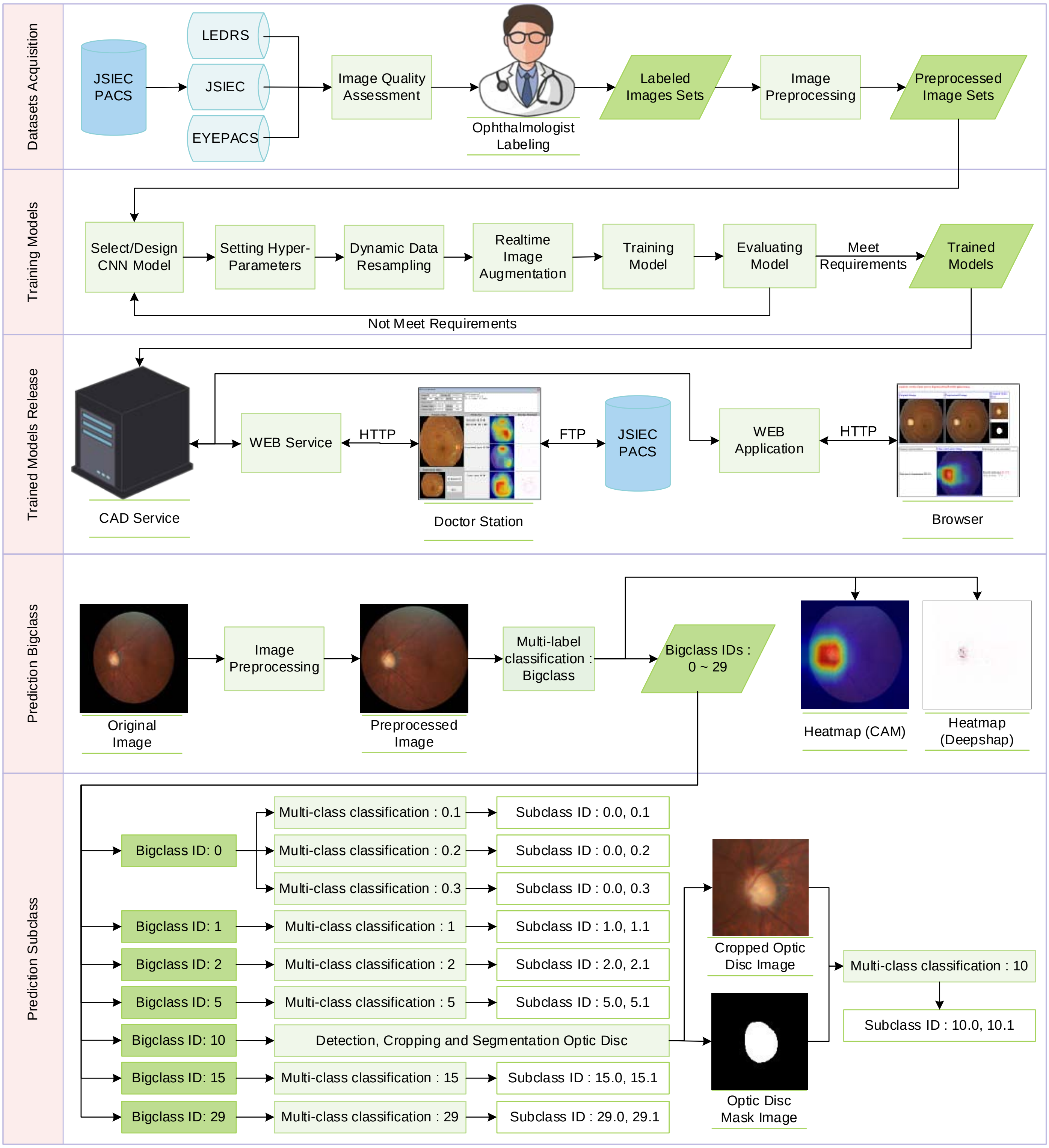

Supplementary Fig. 1 | Architecture, training, and prediction flow of the DLP

Dataset acquisition, model training and release are shown in the three upper rows of the flowchart. Images collected from JSIEC, LEDRS and EYEPACS were firstly filtered by automatic quality assessment, followed by ophthalmologist labeling and preprocessing. Preprocessed image datasets were applied for training and validation of deep learning algorithm models. Real time data augmentation and dynamic resampling were applied during the training procedures. After training and validation, the DLP was deployed as both a web site and a few web services, JSIEC PACS was integrated with the DLP though web services for internal testing. Predictions of bigclasses and subclasses are shown in the bottom. After preprocessing, the preprocessed images were further classified into 30 bigclasses (ID 0~29) with generated heatmaps (CAM and Deepshap). Images classified as bigclass ID 0, 1, 2, 5, 10, 15 and 29 would be further processed with subclass prediction using corresponding models trained independently. There are four

subclasses in bigclass 0. Therefore, three parallel binary classifiers would be applied to detect the probability of the three conditions (Tessellated fundus, large optic cup and DR1) against normal. Images classified as bigclass 10 would be subsequently cropped and segmented into optic disc-centered image with small size (112x112 pixels) for final subclass classification. Flow of the algorithm, is depicted in Supplementary Fig. 4.

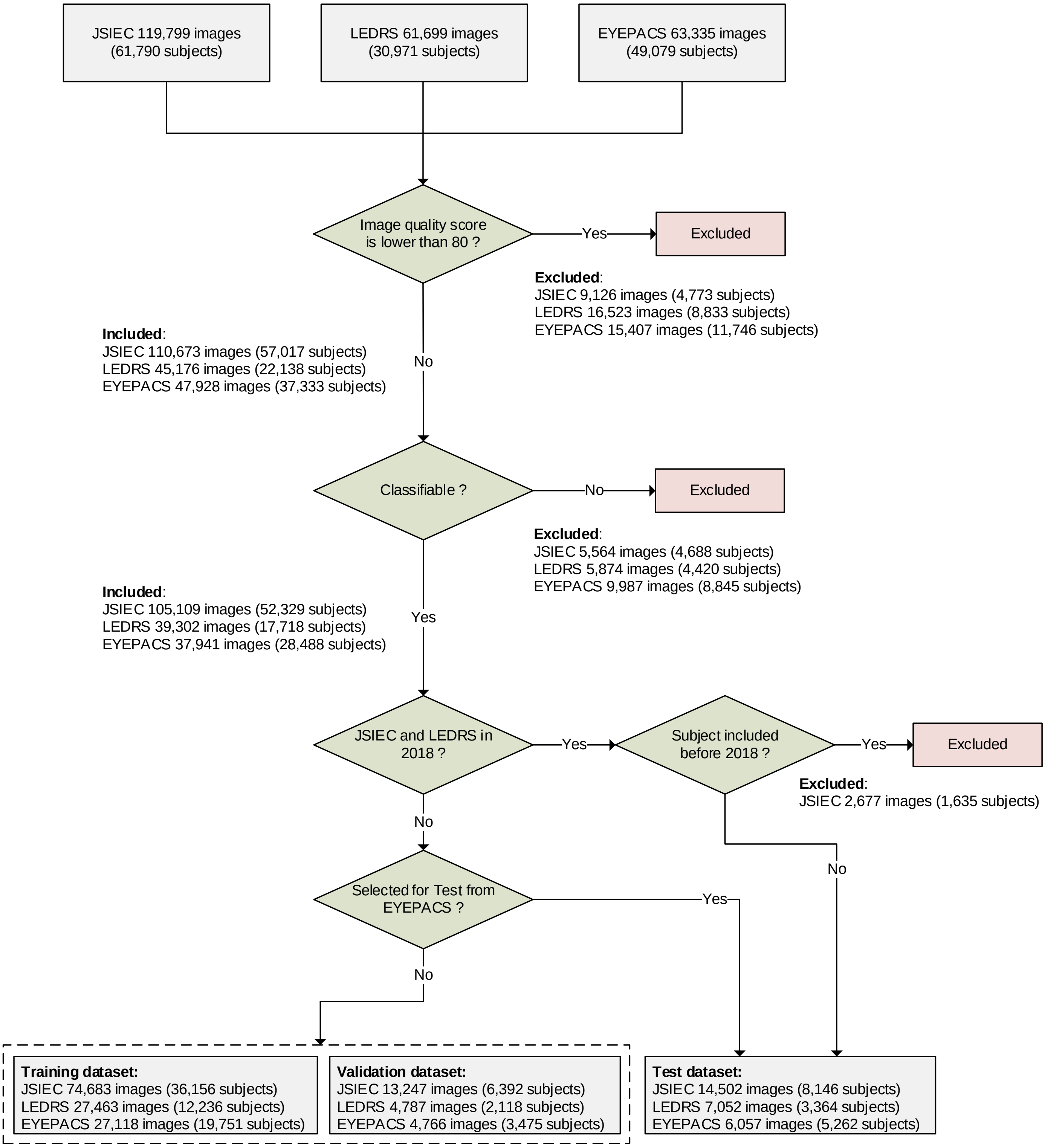

Supplementary Fig. 2 | Sample selection of JSIEC, LEDRS and EYEPACS datasets.

Images collected within year 2018 in JSIEC and LEDRS datasets were applied as test dataset. Patients had been imaged before 2018 were excluded from the dataset of 2018. Images in EYEPACS dataset were randomly split into training, validation and test dataset due to the lack of collection date information.

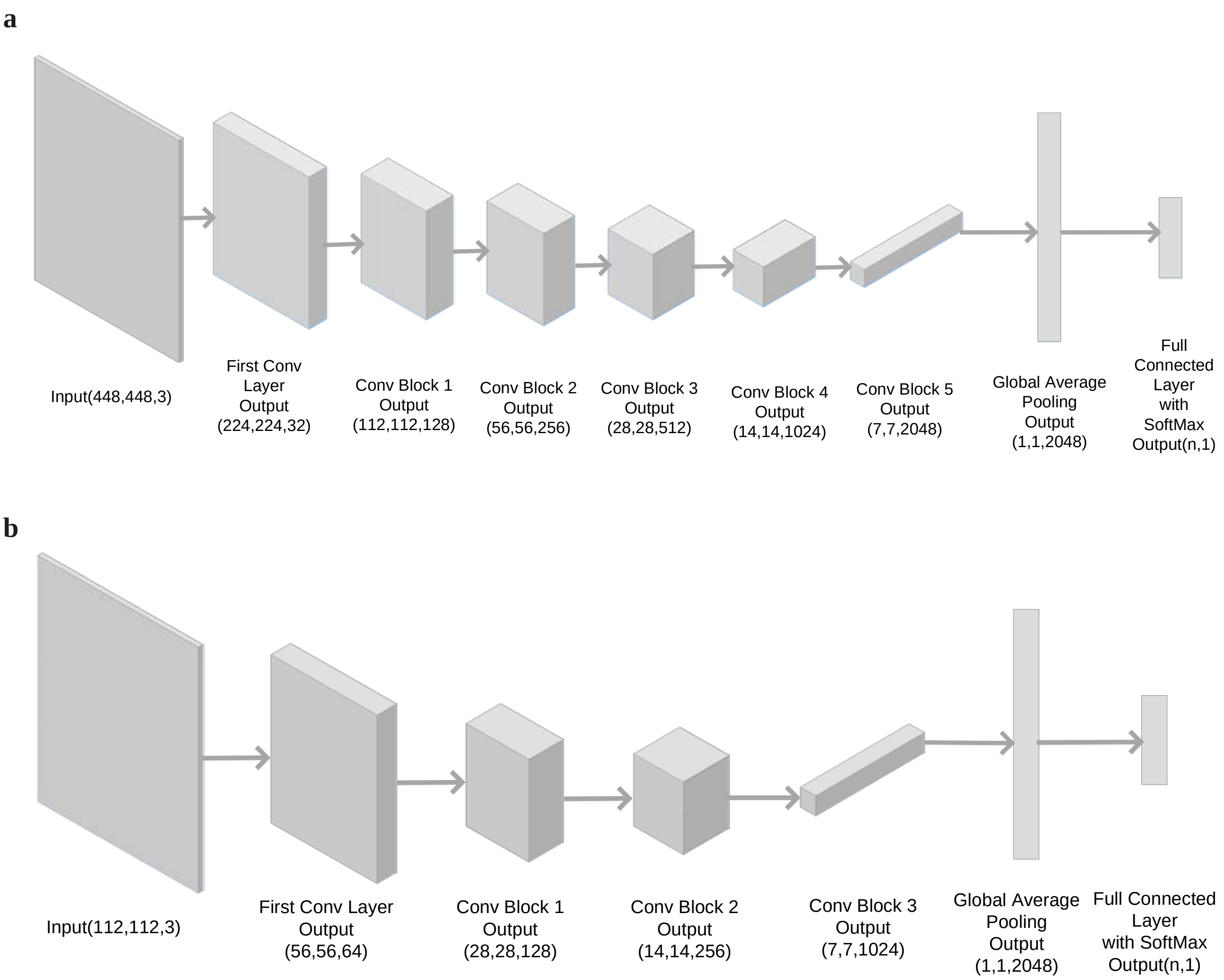

Supplementary Fig. 3 | Architecture of our custom designed CNNs.

(a) Architecture of our custom CNN in group B, base on ResNet and ResNeXt, input image resolution 448x448, used to divide bigclass 0 into normal and DR1. (b) Architecture of our custom CNN in group C, base on ResNet and ResNeXt, input image resolution 112x112, used to divide bigclass 10 into possible glaucoma and optic atrophy.

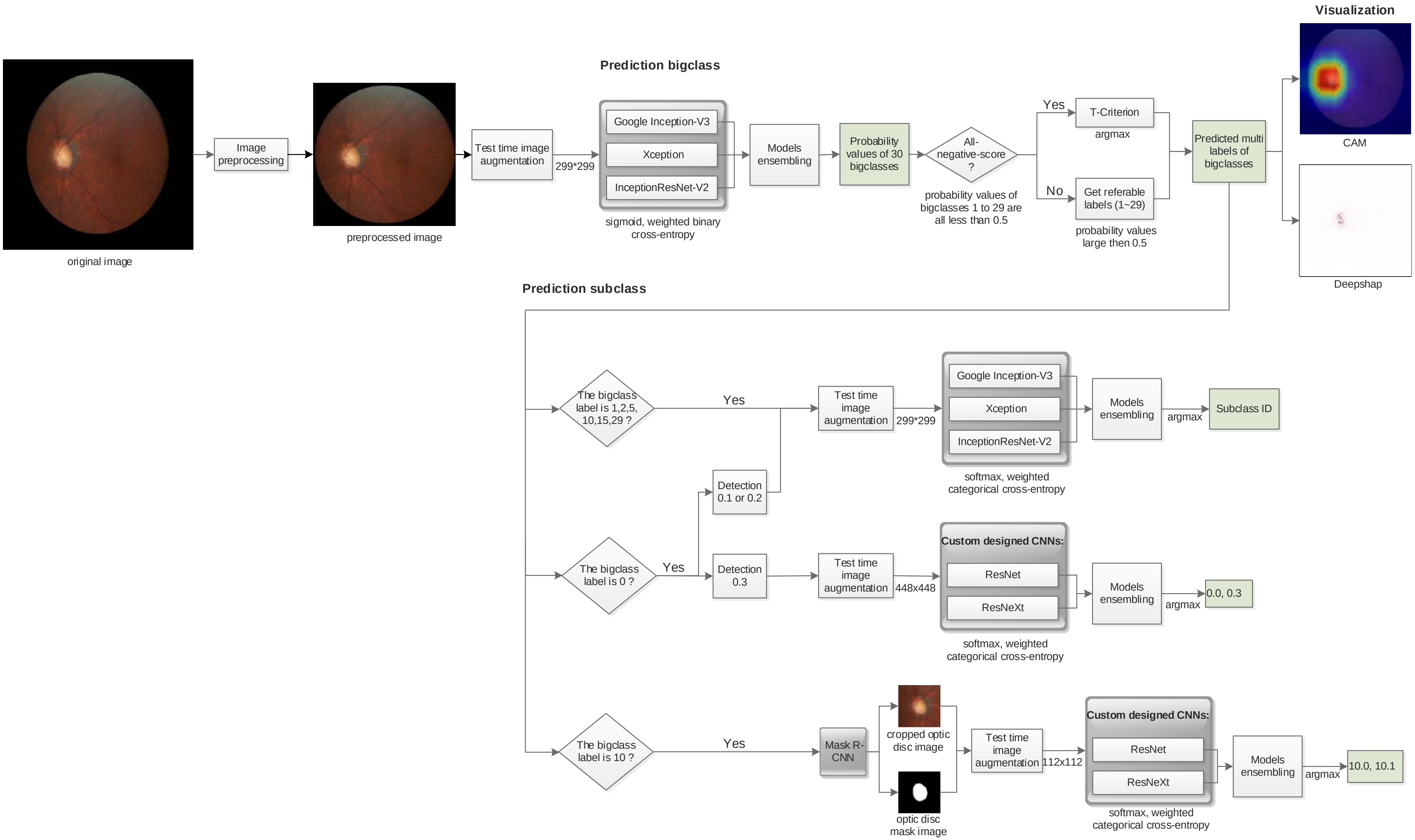

Supplementary Fig. 4 | Classification flow of DLP.

Images classified as bigclass 0, 1, 2, 5, 15 and 29 were further processed with subclass prediction with CNNs trained independently. Images classified as bigclass 10 were subsequently cropped and segmented into optic disc-centered image with small size (112x112 pixels) for final subclass classification. There were four subclasses in bigclass 0; therefore, three parallel binary classifiers applied to detect the probability of the three conditions (tessellated fundus, large optic cup and DR1) against normal. To detect microaneurysms (very small red dots), input image resolution 448x448 was used in custom designed CNNs for detection of subclass 0.3.

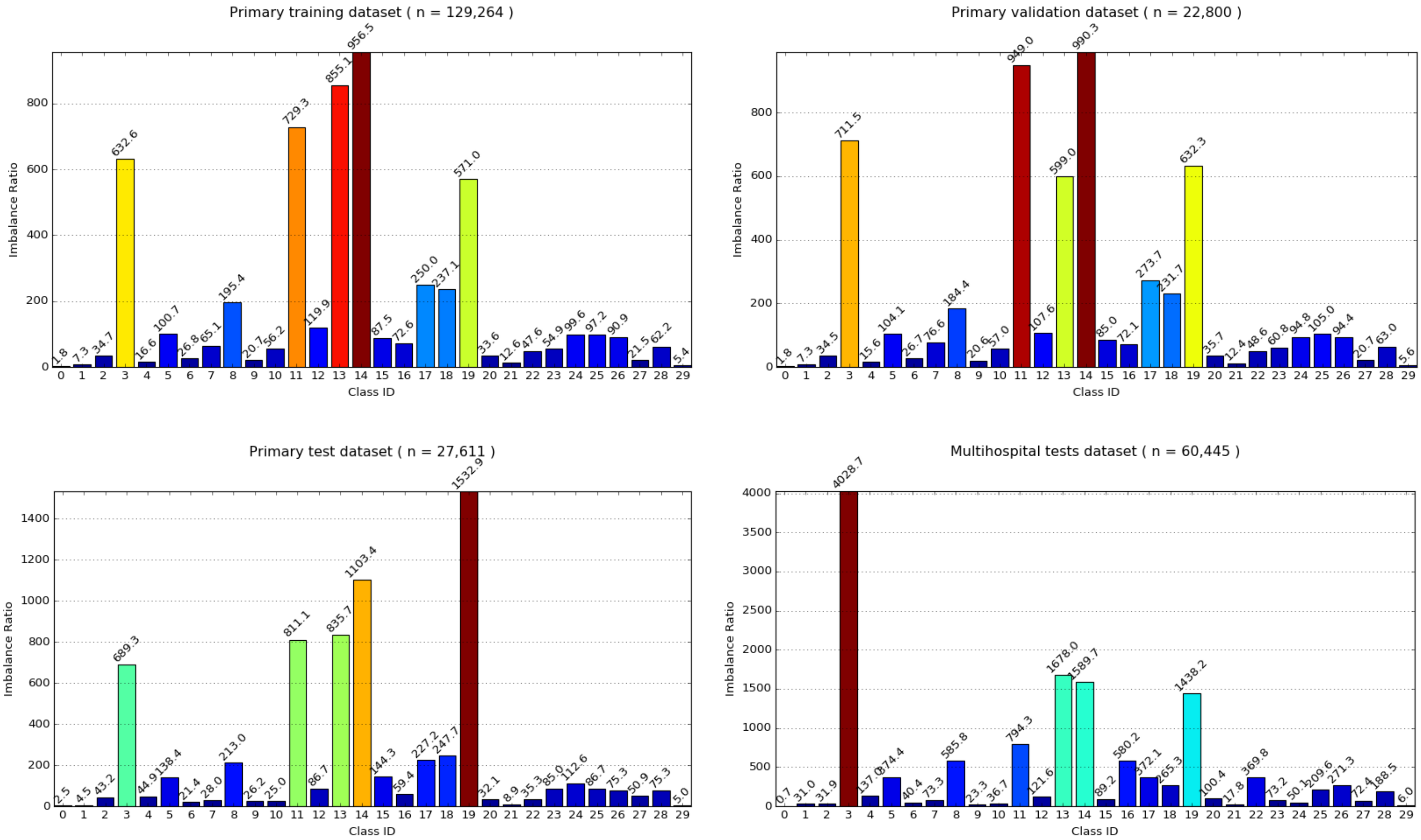

Supplementary Fig. 5 | Imbalance ratio of primary datasets and multihospital tests dataset.
Imbalance ratio = (TN + FP) / (FN + TP)

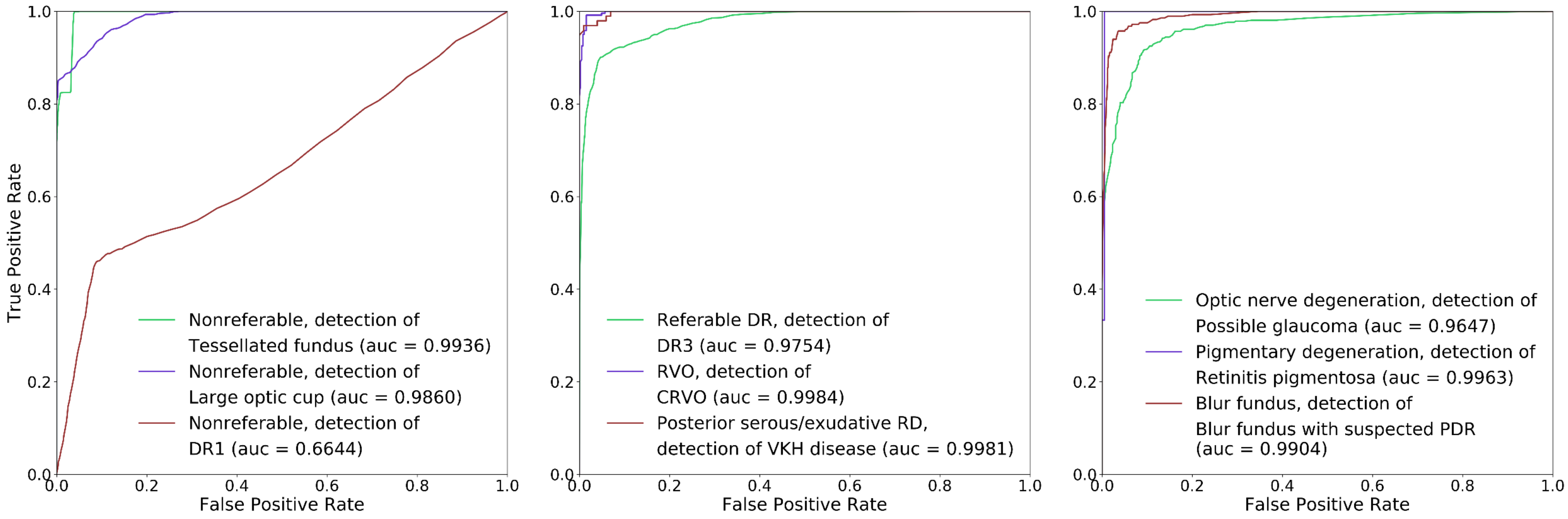

Supplementary Fig. 6 | ROC and AUC of DLP for detection of subclasses in primary test dataset.
Source data are provided as a Source Data file.

a

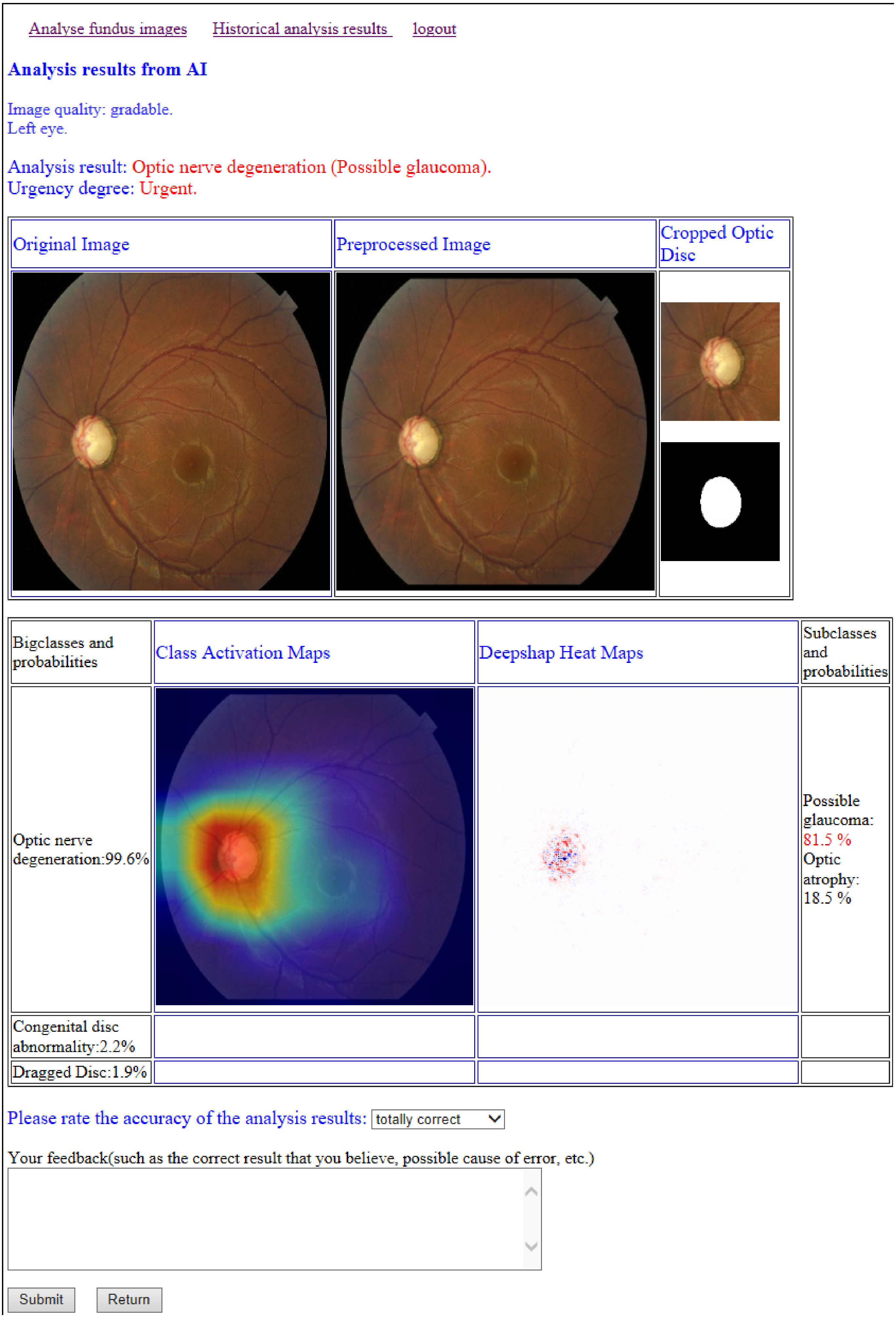
Analyse fundus images
Historical analysis results
logout
Analysis results from AI
Image quality: gradable.
Left eye.
Analysis result: Optic nerve degeneration (Possible glaucoma).
Urgency degree: Urgent.
Original Image
Preprocessed Image
Cropped Optic Disc
Bigclasses and probabilities
Class Activation Maps
Deepshap Heat Maps
Subclasses and probabilities
Optic nerve degeneration:99.6%
Possible glaucoma: 81.5 %
Optic atrophy: 18.5 %
Congenital disc abnormality:2.2%
Dragged Disc:1.9%
Please rate the accuracy of the analysis results:
totally correct
Your feedback(such as the correct result that you believe, possible cause of error, etc.)
Submit
Return

b

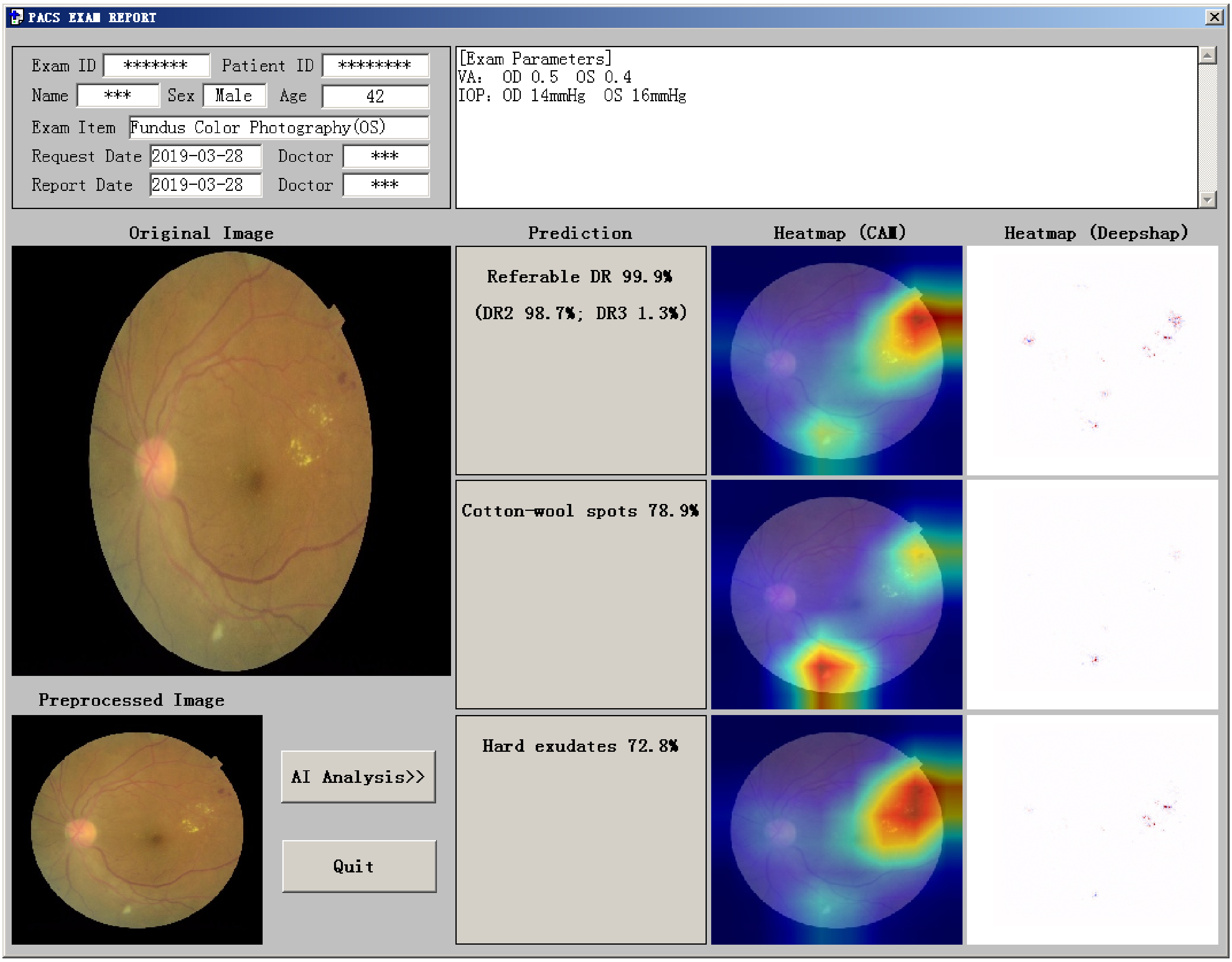

Supplementary Fig. 7 | Screenshot of web-based platform and PACS integration.

a, Web-based platform. An example of detection on a possible glaucoma retinal image with the web-based DLP. Original image, preprocessed image and image of cropped optic disc area and image mask of optic disk are shown on the upper row. Bigclass analyses with probabilities, class activation maps (CAM) and Deepshap are shown on the lower row, with analysis of subclass 10 (optic nerve degeneration) on the right side.

b, Picture archiving and communication systems (PACS) integration. The example was a 42-year-old patient with blurred vision for three months. The patient information and examination details are shown in the top-left column, the examination parameters, such as visual acuity (VA) and intraocular pressure (IOP), are in the top-right column and the original and preprocessed images in the bottom-left column. Three columns on the right show the analyses of every bigclass with probability (subclass if applicable), heatmaps of CAM and Deepshap.

Supplementary Tables

Supplementary Table 1 | Brief descriptions of features in fundus images of diseases and conditions.

ID	Urgency	Diseases/conditions	Brief descriptions of fundus images
0		Nonreferable	
0.0	O	Normal	orange-red fundus with red branched curving vasculature enter the pink optic disc with sharp margins and a C/D ratio of approximately 0.35
0.1	O	Tessellated fundus	diffuse attenuation of the RPE with visibility of large choroidal vessels
0.2	R	Large optic cup	C/D>0.5, with a pink neuroretinal rim in ISNT rule, without notching or bayoneting of vessels
0.3	R	DR1	Microaneurysms only (International Classification of DR 2017)
1		Referable DR	
1.0	S	DR2	microaneurysms and other signs (dot and blot hemorrhages, hard exudates), less than severe nonproliferative DR, and/or with DME
1.1	U	DR3	severe nonproliferative DR and proliferative DR (neovascularization, vitreous/preretinal hemorrhage)
2		RVO	
2.0	S	BRVO	tortuosity and dilatation of affected veins, with dot, blot and flame haemorrhages, sometimes with cotton wool spots or hard exudates
2.1	S	CRVO	tortuosity and dilatation of all branches of veins, with dot, blot and flame haemorrhages, sometimes with cotton wool spots or hard exudates
3	U	RAO	attenuation of arteries and veins, cherry red fovea, in contrast to the cloudy white oedematous retina effected by artery occlusion
4	U	Rhegmatogenous RD	slightly opaque, convex or corrugated appearance of elevated retina, sometimes with breaks in view
5		Posterior serous/exudative RD	
5.0	S	CSCR	round or oval retinal elevation with clear or trubid fluid underneath, sometimes with depigmented RPE foci or small patches of RPE atrophy or hyperplasia
5.1	U	VKH disease	circumscribed retinal edema, multiple exudative retinal detachments of posterior retina, often with optic disc hyperemia and edema, obscure retina with slight radial folds can be seen with the resolving of edema
6	U	Maculopathy	Lesions within macular area, such as intermediate AMD (drusen >125μm), neovascular-AMD, RAP, PCV, CNV, IMT, and macular atrophy, not caused by other listed categories of diseases
7	S	ERM	a cellophane sheen sheet on or above the surface of the retina with macular pucker, distortion of blood vessels within vessel arches
8	U	MH	central foveal defect , round or oval shape, maybe with multiple yellow deposits within the crater surrounded or a cuff of subretinal fluid
9	S	Pathological myopia	tessellated fundus with focal chorioretinal atrophy, fuchs spot, lacquer cracks, CNV or subretinal haemorrhage
10		Optic nerve degeneration	
10.0	U	Possible glaucoma	large C/D ratio with cup excavation, thinning of neuroretinal rim, notching and bayoneting of vessels with RNFL defects, disc haemorrhages, baring of circumlinear blood vessels, laminar dot sign, peripapillary atrophy
10.1	S	Optic atrophy	white disc, reduction of small vessels on the disc, attenuation of peripapillary vessels and thinning of RNFL, sometimes with Paton lines
11	U	Severe hypertensive retinopathy	cotton-wool spots, arteriolar narrowing, arteriolosclerosis, flame-shaped haemorrhages, retinal oedema, macrlar star and disc oedema
12	U	Disc swelling and elevation	disc hyperaemia, elevation of indistinct disc margins, sometimes with peripapillary flame haemorrhages and cotton wool spots
13	R	Dragged disc	temporal vascular straightening, retinal fold or vitreous bands extending from peripheral area to the disc
14	R	Congenital disc abnormality	optic disc coloboma, morning glory anomaly, pit, megalopapilla and hypoplastic disc
15		Pigmentary degeneration	
15.0	R	Retinitis pigmentosa	mid-peripheral RPE atrophy with bone-spicule perivascular pigmentation, arteriorlar attenuation and waxy disc pallor
15.1	R	Bietti crystalline dystrophy	numerous fine, glistening, yellow-white crystals, atrophy of the RPE and choriocapillaris with normal optic disc and retinal vasculature
16	S	Peripheral retinal degeneration and break	Lattice, snailtrack, pavingstone, honeycomb, peripheral drusen, microcystoid and white-without pressure, sometimes with retinal break
17	R	Myelinated nerve fiber	whitish striated patches with feathery borders that obscure retinal vessels
18	S	Vitreous particles	including asteroid hyalosis, synchysis scintillans and deposits on familial amyloidosis
19	U	Fundus neoplasm	slightly elevated, dome or mushroom shaped mass in various colors
20	S	Massive hard exudates	waxy yellow lesions with distinct margins arranged in large clumps, usually caused by vessel abnormalities
21	S	Yellow-white spots/flecks	multiple, discrete, yellow-white, round dot or polymorphous fleck lesions, including early AMD (drusen <125μm)
22	S	Cotton-wool spots	small, whitish, fluffy superficial lesions in the post-equatorial fundus
23	S	Vessel tortuosity	tortuous and sometimes dilated arteries and veins locally or spread the retina
24	S	Chorioretinal atrophy/coloboma	focal or extensive RPE and choroidal atrophy or coloboma
25	U	Preretinal haemorrhage	usually round red lesion obscures all underlying retinal landmarks, sometimes with boat-shaped crescentic configuration, haemorrhage may break though into the vitreous
26	U	Fibrosis	irregular greyish-white opacification often with distortion of the retinal vasculature, crossing vessel arches
27	R	Laser spots	multiple, uniform, round, discrete yellow-white or brown lesions caused by photocoagulation
28	R	Silicon oil in eye	shiny reflection from the retina-oil interface
29		Blur fundus	
29.0	S/P	Blur fundus without PDR	blur retinal landmarks caused by severe lens opacities, vitreous opacities or haemorrhage without PDR
29.1	U/P	Blur fundus with suspected PDR	blur retinal landmarks with suspected features of PDR

Abbreviations: C/D cup disc ratio, DR diabetic retinopathy, PDR proliferative diabetic retinopathy, DME diabetic macular edema, BRVO branch retinal vein occlusion, CRVO central retinal vein occlusion, RAO retinal artery occlusion, RD retinal detachment, CSCR central serous chorioretinopathy, ERM epiretinal membrane, MH macular hole, RPE retinal pigment epithelium, AMD age-related macular degeneration, PCV polypoidal choroidal vasculopathy, CNV choroidal

neovascularization, IMT idiopathic macular telangiectasis, RNFL retinal nerve fiber layer. Annotations: Lattice - spindle shaped areas with arborizing network of white lines and RPE hyperplasia, Snailtrack -sharply demarcated bands of tightly packed snowflakes, Pavingstone - discrete, yellow-white patches of focal chorioretinal atrophy, Honeycomb - fine network of pigmentation, Peripheral drusen - multitude of tiny pale dots that may be associated with mild pigmentary changes, Microcystoid - tiny vesicles with indistinct borders on a greyish-white background, Whit-without pressure - superficial grey area with a geographic configuration. O observation, R routine, S semi-urgent, U urgent, P repeat photography

Supplementary Table 2 | Agreement of labels amongst unspecialized ophthalmologists, senior retina specialists and retina expert panel.

Datasets	Labelled images, No.	Unspecialized ophthalmologists *vs* senior retina specialists, %		Images transferred to retina expert panel, No. [c]	Unspecialized ophthalmologists *vs* retina expert panel, %		Senior retina specialists *vs* retina expert panel, %	
		Subset accuracy [a]	Jaccard index [b]		Subset accuracy	Jaccard index	Subset accuracy	Jaccard index
JSIEC	102,432	84.89	89.33	15,476	28.78	48.37	53.03	69.96
LEDRS	39,302	84.72	87.23	6,006	26.58	35.57	52.07	60.97
EYEPACS	37,941	87.48	89.64	4,748	34	43.81	46.9	54.74
Fujian	39,671	85.05	87.37	5,931	27.4	35.2	49.87	57.59
Tibet	14,826	86.55	90.16	1,993	31.54	47.46	51.01	65.75
Xinjiang	5,948	87.19	89.5	761	25.55	36.1	54.26	62.71

a. Subset accuracy provided the average percentages of samples having identical labels among ophthalmologists of different level of experiences.

b. Jaccard index, also known as Jaccard similarity coefficient, were applied to compare similarities between finite datasets[39]. For the same sample, the label sets (multiple labels) marked by two graders are set A and B, then Jaccard index can be calculated by this formula: $J(A, B) = |A \cap B| / |A \cup B|$.

c. Images without identical labels between unspecialized ophthalmologists and retina specialists were transferred to the retina expert panel.

Supplementary Table 3 | Summary of unclassifiable images judged by ophthalmologists.

Datasets	No. of unclassifiable images		
	Agreement of unspecialized ophthalmologists & senior retina specialists (%) [a]	Retina expert panel (%)	Total unclassifiable images
JSIEC	4,353 (78.2)	1,211 (21.8)	5,564
LEDRS	5,144 (87.6)	730 (12.4)	5,874
EYEPACS	8,633 (86.4)	1,354 (13.6)	9,987
**Total**	18,130 (84.6)	3,295 (15.4)	21,425

a. All images with unclassifiable agreement were also sent to the retina expert panel for final confirmation.

Supplementary Table 4 | Information of classifiers.

Classifier	Classification task	No. of classes	CNNs group	Input image	Input image resolution	Architecture of CNNs model	Training mode	Output
Bigclass	Multi-label classification of 30 bigclasses	30	A	Preprocessed	299x299	Google Inception-V3 & Xception & InceptionResNet-V2	Transfer learning from ImageNet	0 ~ 29, CAM
0.1	Multi-class classification of detection subclass 0.1 in bigclass 0	2	A	Preprocessed	299x299	Google Inception-V3 & Xception & InceptionResNet-V2	Transfer learning from bigclass	0.0, 0.1
0.2	Multi-class classification of detection subclass 0.2 in bigclass 0	2	A	Preprocessed	299x299	Google Inception-V3 & Xception & InceptionResNet-V2	Transfer learning from bigclass	0.0, 0.2
0.3	Multi-class classification of detection subclass 0.3 in bigclass 0	2	B	Preprocessed	448x448	Custom Designed ResNet & ResNeXt	Trained from scratch	0.0, 0.3
1	Multi-class classification of detection subclass 1.1 in bigclass 1	2	A	Preprocessed	299x299	Google Inception-V3 & Xception & InceptionResNet-V2	Transfer learning from bigclass	1.0, 1.1
2	Multi-class classification of detection subclass 2.1 in bigclass 2	2	A	Preprocessed	299x299	Google Inception-V3 & Xception & InceptionResNet-V2	Transfer learning from bigclass	2.0, 2.1
5	Multi-class classification of detection subclass 5.1 in bigclass 5	2	A	Preprocessed	299x299	Google Inception-V3 & Xception & InceptionResNet-V2	Transfer learning from bigclass	5.0, 5.1
10	Multi-class classification of detection subclass 10.1 in bigclass 10	2	C	Preprocessed	384x384 112x112	Mask R-CNN, Custom Designed ResNet & ResNeXt	Trained from scratch	10.0, 10.1
15	Multi-class classification of detection subclass 15.1 in bigclass 15	2	A	Preprocessed	299x299	Google Inception-V3 & Xception & InceptionResNet-V2	Transfer learning from bigclass	15.0, 15.1
29	Multi-class classification of detection subclass 29.1 in bigclass 29	2	A	Preprocessed	299x299	Google Inception-V3 & Xception & InceptionResNet-V2	Transfer learning from bigclass	29.0, 29.1

Supplementary Table 5 | Performance of DLP for detection of bigclasses in primary training dataset (*n*=129,264).

		No. of images							
**Diseases / conditions**	**ID**	**TN**	**FP**	**FN**	**TP**	**F_1**	**Sensitivity**	**Specificity**	**AUC (95% CI)**
Nonreferable	0	83,506	250	3,696	41,812	0.955	0.919	0.997	0.9914 (0.9910-0.9917)
Referable DR	1	112,598	1,076	459	15,131	0.952	0.971	0.991	0.9974 (0.9971-0.9977)
RVO	2	125,598	43	100	3,523	0.980	0.972	1.000	0.9996 (0.9993-0.9999)
RAO	3	129,057	3	1	203	0.990	0.995	1.000	1.0000 (1.0000-1.0000)
Rhegmatogenous RD	4	121,805	120	56	7,283	0.988	0.992	0.999	0.9998 (0.9997-1.0000)
Posterior serous/exudative RD	5	127,729	264	21	1,250	0.898	0.983	0.998	0.9999 (0.9998-0.9999)
Maculopathy	6	124,452	164	140	4,508	0.967	0.970	0.999	0.9994 (0.9991-0.9997)
ERM	7	126,814	494	80	1,876	0.867	0.959	0.996	0.9968 (0.9954-0.9981)
MH	8	128,523	83	12	646	0.932	0.982	0.999	0.9996 (0.9990-1.0000)
Pathological myopia	9	123,151	168	27	5,918	0.984	0.995	0.999	1.0000 (0.9999-1.0000)
Optic nerve degeneration	10	126,286	719	27	2,232	0.857	0.988	0.994	0.9988 (0.9984-0.9993)
Severe hypertensive retinopathy	11	129,040	47	4	173	0.872	0.977	1.000	1.0000 (0.9999-1.0000)
Disc swelling and elevation	12	128,116	79	12	1,057	0.959	0.989	0.999	1.0000 (0.9999-1.0000)
Dragged disc	13	129,104	9	1	150	0.968	0.993	1.000	1.0000 (1.0000-1.0000)
Congenital disc abnormality	14	129,098	31	0	135	0.897	1.000	1.000	1.0000 (1.0000-1.0000)
Pigmentary degeneration	15	127,691	112	0	1,461	0.963	1.000	0.999	1.0000 (1.0000-1.0000)
Peripheral retinal degeneration and break	16	126,878	630	14	1,742	0.844	0.992	0.995	0.9995 (0.9994-0.9996)
Myelinated nerve fiber	17	128,747	2	2	513	0.996	0.996	1.000	1.0000 (1.0000-1.0000)
Vitreous particles	18	128,592	129	0	543	0.894	1.000	0.999	1.0000 (1.0000-1.0000)
Fundus neoplasm	19	129,012	26	1	225	0.943	0.996	1.000	1.0000 (1.0000-1.0000)
Hard exudates	20	124,952	579	13	3,720	0.926	0.997	0.995	0.9989 (0.9988-0.9990)
Yellow-white spots/flecks	21	117,755	1,978	54	9,477	0.903	0.994	0.983	0.9984 (0.9982-0.9986)
Cotton-wool spots	22	126,431	172	111	2,550	0.947	0.958	0.999	0.9987 (0.9983-0.9991)
Vessel tortuosity	23	126,442	511	8	2,303	0.899	0.997	0.996	0.9990 (0.9989-0.9992)
Chorioretinal atrophy/coloboma	24	127,633	346	4	1,281	0.880	0.997	0.997	0.9999 (0.9999-0.9999)
Preretinal haemorrhage	25	127,283	665	19	1,297	0.791	0.986	0.995	0.9991 (0.9989-0.9994)
Fibrosis	26	127,510	347	83	1,324	0.860	0.941	0.997	0.9984 (0.9977-0.9991)
Laser spots	27	123,403	122	7	5,732	0.989	0.999	0.999	1.0000 (1.0000-1.0000)
Silicon oil in eye	28	127,079	140	15	2,030	0.963	0.993	0.999	1.0000 (1.0000-1.0000)
Blur fundus	29	107,664	1,475	131	19,994	0.961	0.993	0.986	0.9995 (0.9994-0.9996)
**Referable, frequency-weighted average**						**0.946**	**0.965**	**0.997**	**0.9994**
**Subset accuracy, %**		**91.32**							

Supplementary Table 6 | Performance of DLP for detection of bigclasses in primary validation dataset (*n*=22,800).

Diseases / conditions	ID	No. of images TN	FP	FN	TP	F_1	Sensitivity	Specificity	AUC (95% CI)
Nonreferable	0	14,726	40	669	7,365	0.954	0.917	0.997	0.9923 (0.9915-0.9931)
Referable DR	1	19,882	184	91	2,643	0.951	0.967	0.991	0.9972 (0.9964-0.9980)
RVO	2	22,148	10	20	622	0.976	0.969	1.000	0.9999 (0.9998-0.9999)
RAO	3	22,768	0	0	32	1.000	1.000	1.000	1.0000 (1.0000-1.0000)
Rhegmatogenous RD	4	21,408	19	11	1,362	0.989	0.992	0.999	0.9999 (0.9999-1.0000)
Posterior serous/exudative RD	5	22,534	49	5	212	0.887	0.977	0.998	0.9998 (0.9997-0.9999)
Maculopathy	6	21,948	30	32	790	0.962	0.961	0.999	0.9988 (0.9977-0.9999)
ERM	7	22,431	75	21	273	0.850	0.929	0.997	0.9905 (0.9834-0.9977)
MH	8	22,660	17	2	121	0.927	0.984	0.999	0.9999 (0.9998-1.0000)
Pathological myopia	9	21,711	33	7	1,049	0.981	0.993	0.998	0.9998 (0.9996-1.0000)
Optic nerve degeneration	10	22,277	130	7	386	0.849	0.982	0.994	0.9988 (0.9983-0.9993)
Severe hypertensive retinopathy	11	22,761	15	1	23	0.742	0.958	0.999	0.9999 (0.9998-1.0000)
Disc swelling and elevation	12	22,572	18	3	207	0.952	0.986	0.999	0.9999 (0.9997-1.0000)
Dragged disc	13	22,759	3	0	38	0.962	1.000	1.000	1.0000 (1.0000-1.0000)
Congenital disc abnormality	14	22,774	3	0	23	0.939	1.000	1.000	1.0000 (1.0000-1.0000)
Pigmentary degeneration	15	22,508	27	0	265	0.952	1.000	0.999	1.0000 (1.0000-1.0000)
Peripheral retinal degeneration and break	16	22,364	124	2	310	0.831	0.994	0.994	0.9994 (0.9991-0.9997)
Myelinated nerve fiber	17	22,716	1	2	81	0.982	0.976	1.000	1.0000 (1.0000-1.0000)
Vitreous particles	18	22,683	19	0	98	0.912	1.000	0.999	1.0000 (1.0000-1.0000)
Fundus neoplasm	19	22,760	4	0	36	0.947	1.000	1.000	1.0000 (1.0000-1.0000)
Hard exudates	20	22,059	120	3	618	0.909	0.995	0.995	0.9986 (0.9983-0.9990)
Yellow-white spots/flecks	21	20,731	363	7	1,699	0.902	0.996	0.983	0.9984 (0.9982-0.9987)
Cotton-wool spots	22	22,313	27	18	442	0.952	0.961	0.999	0.9985 (0.9973-0.9996)
Vessel tortuosity	23	22,308	123	4	365	0.852	0.989	0.995	0.9988 (0.9985-0.9991)
Chorioretinal atrophy/coloboma	24	22,499	63	0	238	0.883	1.000	0.997	0.9999 (0.9999-1.0000)
Preretinal haemorrhage	25	22,469	116	2	213	0.783	0.991	0.995	0.9994 (0.9991-0.9997)
Fibrosis	26	22,495	66	9	230	0.860	0.962	0.997	0.9984 (0.9964-1.0000)
Laser spots	27	21,730	19	1	1,050	0.991	0.999	0.999	1.0000 (1.0000-1.0000)
Silicon oil in eye	28	22,417	27	1	355	0.962	0.997	0.999	1.0000 (0.9999-1.0000)
Blur fundus	29	19,048	279	20	3,453	0.959	0.994	0.986	0.9995 (0.9993-0.9997)
**Referable, frequency-weighted average**						**0.944**	**0.963**	**0.997**	**0.9994**
**Subset accuracy, %**		**90.77**							

Supplementary Table 7 | Performance of DLP for detection of bigclasses in primary test dataset (*n*=27,611).

		No. of images							
**Diseases / conditions**	**ID**	**TN**	**FP**	**FN**	**TP**	**F_1**	**Sensitivity**	**Specificity**	**AUC (95% CI)**
Nonreferable	0	19,572	108	810	7,121	0.939	0.898	0.995	0.9888 (0.9878-0.9898)
Referable DR	1	22,130	486	154	4,841	0.938	0.969	0.979	0.9936 (0.9928-0.9945)
RVO	2	26,979	7	14	611	0.983	0.978	1.000	0.9985 (0.9965-1.0000)
RAO	3	27,568	3	2	38	0.938	0.950	1.000	1.0000 (0.9999-1.0000)
Rhegmatogenous RD	4	26,954	56	7	594	0.950	0.988	0.998	0.9997 (0.9994-0.9999)
Posterior serous/exudative RD	5	27,334	79	2	196	0.829	0.990	0.997	0.9994 (0.9985-1.0000)
Maculopathy	6	26,319	57	30	1,205	0.965	0.976	0.998	0.9991 (0.9989-0.9994)
ERM	7	26,428	230	40	913	0.871	0.958	0.991	0.9972 (0.9964-0.9980)
MH	8	27,463	19	2	127	0.924	0.984	0.999	0.9997 (0.9994-1.0000)
Pathological myopia	9	26,476	120	3	1,012	0.943	0.997	0.995	0.9997 (0.9996-0.9998)
Optic nerve degeneration	10	26,191	357	9	1,054	0.852	0.992	0.987	0.9964 (0.9958-0.9969)
Severe hypertensive retinopathy	11	27,563	14	0	34	0.829	1.000	0.999	0.9993 (0.9990-0.9996)
Disc swelling and elevation	12	27,225	71	1	314	0.897	0.997	0.997	0.9997 (0.9995-0.9998)
Dragged disc	13	27,570	8	0	33	0.892	1.000	1.000	0.9999 (0.9998-1.0000)
Congenital disc abnormality	14	27,581	5	0	25	0.909	1.000	1.000	0.9999 (0.9997-1.0000)
Pigmentary degeneration	15	27,378	43	0	190	0.898	1.000	0.998	0.9999 (0.9999-1.0000)
Peripheral retinal degeneration and break	16	27,018	136	0	457	0.870	1.000	0.995	0.9997 (0.9996-0.9998)
Myelinated nerve fiber	17	27,484	6	6	115	0.950	0.950	1.000	0.9956 (0.9873-1.0000)
Vitreous particles	18	27,475	25	0	111	0.899	1.000	0.999	1.0000 (0.9999-1.0000)
Fundus neoplasm	19	27,589	4	0	18	0.900	1.000	1.000	0.9999 (0.9998-1.0000)
Hard exudates	20	26,645	133	1	832	0.925	0.999	0.995	0.9989 (0.9985-0.9993)
Yellow-white spots/flecks	21	24,384	436	68	2,723	0.915	0.976	0.982	0.9927 (0.9910-0.9944)
Cotton-wool spots	22	26,764	87	12	748	0.938	0.984	0.997	0.9987 (0.9981-0.9993)
Vessel tortuosity	23	27,231	59	6	315	0.906	0.981	0.998	0.9996 (0.9994-0.9997)
Chorioretinal atrophy/coloboma	24	27,308	60	14	229	0.861	0.942	0.998	0.9962 (0.9921-1.0000)
Preretinal haemorrhage	25	27,105	191	1	314	0.766	0.997	0.993	0.9985 (0.9979-0.9991)
Fibrosis	26	27,164	85	2	360	0.892	0.994	0.997	0.9992 (0.9988-0.9995)
Laser spots	27	27,068	11	24	508	0.967	0.955	1.000	0.9996 (0.9994-0.9997)
Silicon oil in eye	28	27,225	24	3	359	0.964	0.992	0.999	0.9991 (0.9974-1.0000)
Blur fundus	29	22,532	475	101	4,503	0.940	0.978	0.979	0.9961 (0.9953-0.9970)
**Referable, frequency-weighted average**						**0.923**	**0.978**	**0.996**	**0.9984**
**Subset accuracy, %**		**87.98**							

Supplementary Table 8 | Performance of DLP for detection of bigclasses in multihospital tests dataset (*n*=60,445).

		No. of images							
**Diseases / conditions**	**ID**	**TN**	**FP**	**FN**	**TP**	**F_1**	**Sensitivity**	**Specificity**	**AUC (95% CI)**
Nonreferable	0	25,417	330	2,019	32,679	0.965	0.942	0.987	0.9765 (0.9754-0.9775)
Referable DR	1	58,373	182	35	1,855	0.945	0.981	0.997	0.9987 (0.9984-0.9990)
RVO	2	58,576	29	80	1,760	0.970	0.957	1.000	0.9995 (0.9992-0.9998)
RAO	3	60,429	1	1	14	0.933	0.933	1.000	0.9999 (0.9997-1.0000)
Rhegmatogenous RD	4	59,938	69	3	435	0.924	0.993	0.999	0.9998 (0.9998-0.9999)
Posterior serous/exudative RD	5	60,202	82	1	160	0.794	0.994	0.999	0.9996 (0.9995-0.9998)
Maculopathy	6	58,903	83	80	1,379	0.944	0.945	0.999	0.9990 (0.9988-0.9992)
ERM	7	59,382	250	50	763	0.836	0.938	0.996	0.9976 (0.9969-0.9982)
MH	8	60,334	8	6	97	0.933	0.942	1.000	0.9996 (0.9991-1.0000)
Pathological myopia	9	57,721	240	24	2,460	0.949	0.990	0.996	0.9994 (0.9993-0.9995)
Optic nerve degeneration	10	58,336	504	68	1,537	0.843	0.958	0.991	0.9972 (0.9967-0.9977)
Severe hypertensive retinopathy	11	60,365	4	11	65	0.897	0.855	1.000	0.9998 (0.9997-0.9999)
Disc swelling and elevation	12	59,821	131	10	483	0.873	0.980	0.998	0.9992 (0.9989-0.9995)
Dragged disc	13	60,397	12	0	36	0.857	1.000	1.000	0.9999 (0.9999-1.0000)
Congenital disc abnormality	14	60,397	10	0	38	0.884	1.000	1.000	0.9998 (0.9995-1.0000)
Pigmentary degeneration	15	59,663	112	7	663	0.918	0.990	0.998	0.9995 (0.9992-0.9999)
Peripheral retinal degeneration and break	16	60,230	111	0	104	0.652	1.000	0.998	0.9995 (0.9993-0.9997)
Myelinated nerve fiber	17	60,280	3	12	150	0.952	0.926	1.000	0.9998 (0.9996-0.9999)
Vitreous particles	18	60,188	30	1	226	0.936	0.996	1.000	1.0000 (0.9999-1.0000)
Fundus neoplasm	19	60,392	11	3	39	0.848	0.929	1.000	0.9999 (0.9998-1.0000)
Hard exudates	20	59,752	97	3	593	0.922	0.995	0.998	0.9996 (0.9994-0.9997)
Yellow-white spots/flecks	21	56,751	482	133	3,079	0.909	0.959	0.992	0.9969 (0.9965-0.9974)
Cotton-wool spots	22	60,196	86	11	152	0.758	0.933	0.999	0.9988 (0.9983-0.9993)
Vessel tortuosity	23	59,499	131	47	768	0.896	0.942	0.998	0.9985 (0.9982-0.9988)
Chorioretinal atrophy/coloboma	24	59,096	166	79	1,104	0.900	0.933	0.997	0.9976 (0.9968-0.9983)
Preretinal haemorrhage	25	59,989	169	4	283	0.766	0.986	0.997	0.9990 (0.9983-0.9998)
Fibrosis	26	60,130	93	3	219	0.820	0.986	0.998	0.9995 (0.9993-0.9997)
Laser spots	27	59,611	10	17	807	0.984	0.979	1.000	0.9999 (0.9999-1.0000)
Silicon oil in eye	28	60,070	56	17	302	0.892	0.947	0.999	0.9969 (0.9928-1.0000)
Blur fundus	29	50,858	908	124	8,555	0.943	0.986	0.982	0.9964 (0.9959-0.9968)
**Referable, frequency-weighted average**						**0.920**	**0.971**	**0.998**	**0.9990**
**Subset accuracy, %**		**92.62**							

Supplementary Table 9 | Performance of DLP for detection of specified diseases in public test datasets (n=3,438).

Datasets	Diseases / conditions	No. of images TN	FP	FN	TP	F_1	Sensitivity	Specificity	AUC (95% CI)
messidor-2	Referable DR	1,340	5	38	365	0.944	0.906	0.996	0.9861 (0.9797-0.9924)
IDRID	Referable DR	174	19	57	266	0.875	0.824	0.902	0.9431 (0.9252-0.9610)
PALM	Pathological myopia	159	2	9	204	0.974	0.958	0.988	0.9931 (0.9870-0.9992)
REFUGE [a]	Optic nerve degeneration	659	61	12	68	0.651	0.850	0.915	0.9397 (0.9065-0.9728)
	Possible glaucoma	672	48	15	65	0.674	0.813	0.933	N/A

a. The REFUGE dataset was applied for detection of glaucoma, which is a subclass of optic nerve degeneration id our DLP. Therefore, we have provided the results for detecting both optic nerve degeneration and possible glaucoma. Results of detecting possible glaucoma were obtained by further classification of optic nerve degeneration FP and TP samples by the subclass algorithm.

Supplementary Table 10 | Performance of DLP for detection of bigclasses in tele-reading categorized (*n*=6,062).

Diseases / conditions	ID	No. of images TN	FP	FN	TP	F_1	Sensitivity	Specificity	AUC (95% CI)
Nonreferable	0	3,477	15	159	2,411	0.965	0.938	0.996	0.9796 (0.9769-0.9823)
Referable DR	1	5,632	22	25	383	0.942	0.939	0.996	0.9864 (0.9802-0.9926)
RVO	2	5,920	5	8	129	0.952	0.942	0.999	0.9983 (0.9971-0.9995)
RAO	3	6,055	1	0	6	0.923	1.000	1.000	0.9996 (0.9990-1.0000)
Rhegmatogenous RD	4	5,996	7	5	54	0.900	0.915	0.999	0.9976 (0.9959-0.9994)
Posterior serous/exudative RD	5	5,953	39	1	69	0.775	0.986	0.993	0.9978 (0.9965-0.9991)
Maculopathy	6	5,966	3	13	80	0.909	0.860	0.999	0.9939 (0.9905-0.9973)
ERM	7	5,765	41	16	240	0.894	0.938	0.993	0.9827 (0.9742-0.9911)
MH	8	6,036	3	1	22	0.917	0.957	1.000	0.9994 (0.9987-1.0000)
Pathological myopia	9	5,615	29	12	406	0.952	0.971	0.995	0.9985 (0.9980-0.9991)
Optic nerve degeneration	10	5,675	62	25	300	0.873	0.923	0.989	0.9881 (0.9852-0.9909)
Severe hypertensive retinopathy	11	6,059	0	0	3	1.000	1.000	1.000	0.9998 (0.9994-1.0000)
Disc swelling and elevation	12	6,004	6	0	52	0.945	1.000	0.999	0.9995 (0.9990-1.0000)
Dragged disc	13	6,057	2	0	3	0.750	1.000	1.000	1.0000 (1.0000-1.0000)
Congenital disc abnormality	14	6,054	3	0	5	0.769	1.000	1.000	0.9999 (0.9998-1.0000)
Pigmentary degeneration	15	5,977	18	1	66	0.874	0.985	0.997	0.9975 (0.9957-0.9994)
Peripheral retinal degeneration and break	16	6,057	5	0	0	0.000	N/A	0.999	N/A
Myelinated nerve fiber	17	6,047	1	3	11	0.846	0.786	1.000	0.9997 (0.9994-1.0000)
Vitreous particles	18	5,992	17	2	51	0.843	0.962	0.997	0.9984 (0.9956-1.0000)
Fundus neoplasm	19	6,059	0	1	2	0.800	0.667	1.000	0.9703 (0.9121-1.0000)
Hard exudates	20	5,946	8	0	108	0.964	1.000	0.999	0.9996 (0.9991-1.0000)
Yellow-white spots/flecks	21	5,224	59	36	743	0.940	0.954	0.989	0.9854 (0.9806-0.9902)
Cotton-wool spots	22	6,012	8	3	39	0.876	0.929	0.999	0.9987 (0.9974-1.0000)
Vessel tortuosity	23	6,005	3	3	51	0.944	0.944	1.000	0.9990 (0.9982-0.9998)
Chorioretinal atrophy/coloboma	24	6,031	3	3	25	0.893	0.893	1.000	0.9986 (0.9972-1.0000)
Preretinal haemorrhage	25	5,987	28	0	47	0.770	1.000	0.995	0.9988 (0.9980-0.9997)
Fibrosis	26	5,988	11	3	60	0.896	0.952	0.998	0.9922 (0.9822-1.0000)
Laser spots	27	6,037	0	1	24	0.980	0.960	1.000	0.9999 (0.9998-1.0000)
Silicon oil in eye	28	6,050	4	1	7	0.737	0.875	0.999	0.9926 (0.9851-1.0000)
Blur fundus	29	5,151	116	44	751	0.904	0.945	0.978	0.9795 (0.9736-0.9854)
**Referable, frequency-weighted average**						**0.913**	**0.948**	**0.997**	**0.9949**
**Subset accuracy, %**		**91.41**							

Supplementary Table 11 | Performance of DLP for detection of subclasses in primary test dataset.

		No. of images							
ID	Diseases / conditions	TN	FP	FN	TP	F_1	Sensitivity	Specificity	AUC (95% CI)
0	Nonreferable, detection of Tessellated fundus	5,461	177	402	1,891	0.867	0.825	0.969	0.9936 (0.9926-0.9946)
0	Nonreferable, detection of Large optic cup	5,636	256	228	1,811	0.882	0.888	0.957	0.9860 (0.9840-0.9879)
0	Nonreferable, detection of DR1	6,083	556	710	582	0.479	0.45	0.916	0.6644 (0.6459-0.6829)
1	Referable DR, detection of DR3	3,657	165	121	1,052	0.88	0.897	0.957	0.9754 (0.9712-0.9796)
2	RVO, detection of CRVO	379	6	5	235	0.977	0.979	0.984	0.9984 (0.9971-0.9998)
5	Posterior serous/exudative RD, detection of VKH disease	96	5	2	95	0.964	0.979	0.95	0.9981 (0.9956-1.0000)
10	Optic nerve degeneration, detection of Possible glaucoma	541	56	38	428	0.901	0.918	0.906	0.9647 (0.9545-0.9750)
15	Pigmentary degeneration, detection of Retinitis pigmentosa	180	1	1	8	0.889	0.889	0.994	0.9963 (0.9889-1.0000)
29	Blur fundus, detection of Blur fundus with suspected PDR	4,214	108	17	265	0.809	0.94	0.975	0.9904 (0.9865-0.9942)

Supplementary Table 12 | Performance of DLP in JSIEC comparative test dataset compared to experts (*n*=711).

		Average expert (fundus only)			Average expert (fundus + note)			DLP (fundus only)			
		F_1	Sensitivity	Specificity	F_1	Sensitivity	Specificity	F_1	Sensitivity	Specificity	AUC (95% CI)
Nonreferable	0	0.890	0.941	0.991	0.936	0.984	0.993	0.986	0.973	1.000	0.9954 (0.9875-1.0000)
Referable DR	1	0.950	0.935	0.991	0.966	0.953	0.994	0.969	0.951	0.996	0.9935 (0.9883-0.9987)
RVO	2	0.954	0.945	0.996	0.957	0.939	0.998	0.977	0.985	0.997	0.9982 (0.9956-1.0000)
RAO	3	0.929	0.920	0.999	0.931	0.940	0.999	1.000	1.000	1.000	1.0000 (1.0000-1.0000)
Rhegmatogenous RD	4	0.985	1.000	0.999	0.985	1.000	0.999	0.962	0.962	0.999	0.9997 (0.9991-1.0000)
Posterior serous/exudative RD	5	0.949	0.936	0.999	0.954	0.945	0.999	0.957	1.000	0.997	0.9988 (0.9972-1.0000)
Maculopathy	6	0.957	0.953	0.996	0.955	0.950	0.996	0.958	0.950	0.997	0.9954 (0.9903-1.0000)
ERM	7	0.967	0.957	0.998	0.972	0.965	0.998	0.968	0.978	0.997	0.9974 (0.9937-1.0000)
MH	8	0.943	0.928	0.999	0.951	0.936	0.999	0.920	0.920	0.997	0.9615 (0.8869-1.0000)
Pathological myopia	9	0.959	0.940	0.998	0.961	0.948	0.998	0.990	0.980	1.000	0.9998 (0.9995-1.0000)
Optic nerve degeneration	10	0.961	0.959	0.998	0.961	0.959	0.998	0.963	1.000	0.996	0.9995 (0.9985-1.0000)
Severe hypertensive retinopathy	11	0.900	0.900	0.997	0.930	0.956	0.997	0.837	1.000	0.990	0.9939 (0.9869-1.0000)
Disc swelling and elevation	12	0.966	0.949	0.999	0.974	0.959	0.999	0.975	1.000	0.997	0.9998 (0.9994-1.0000)
Dragged disc	13	0.937	0.893	1.000	0.952	0.933	0.999	0.966	0.933	1.000	0.9843 (0.9536-1.0000)
Congenital disc abnormality	14	0.849	0.886	0.998	0.925	0.886	1.000	0.923	0.857	1.000	0.9816 (0.9455-1.0000)
Pigmentary degeneration	15	0.982	0.964	1.000	0.982	0.964	1.000	1.000	1.000	1.000	1.0000 (1.0000-1.0000)
Peripheral retinal degeneration and break	16	0.969	0.946	1.000	0.976	0.954	1.000	1.000	1.000	1.000	1.0000 (1.0000-1.0000)
Myelinated nerve fiber	17	0.993	1.000	1.000	0.993	1.000	1.000	1.000	1.000	1.000	1.0000 (1.0000-1.0000)
Vitreous particles	18	1.000	1.000	1.000	1.000	1.000	1.000	0.923	1.000	0.999	1.0000 (1.0000-1.0000)
Fundus neoplasm	19	0.938	1.000	0.999	0.938	1.000	0.999	1.000	1.000	1.000	1.0000 (1.0000-1.0000)
Hard exudates	20	0.979	1.000	0.998	0.982	1.000	0.999	0.966	1.000	0.997	0.9998 (0.9994-1.0000)
Yellow-white spots/flecks	21	0.922	0.932	0.994	0.924	0.936	0.994	0.865	0.957	0.982	0.9897 (0.9818-0.9977)
Cotton-wool spots	22	0.964	0.940	0.998	0.968	0.950	0.998	0.985	0.971	1.000	0.9995 (0.9987-1.0000)
Vessel tortuosity	23	0.824	0.754	0.999	0.852	0.800	0.999	0.870	0.769	1.000	0.9910 (0.9789-1.0000)
Chorioretinal atrophy/coloboma	24	0.934	0.904	0.999	0.931	0.912	0.998	0.926	1.000	0.994	0.9977 (0.9951-1.0000)
Preretinal haemorrhage	25	0.941	0.952	0.997	0.941	0.952	0.997	0.980	0.960	1.000	0.9903 (0.9711-1.0000)
Fibrosis	26	0.956	0.941	0.999	0.953	0.935	0.999	0.974	1.000	0.997	0.9989 (0.9971-1.0000)
Laser spots	27	0.972	0.946	1.000	0.972	0.946	1.000	0.960	0.923	1.000	0.9894 (0.9761-1.0000)
Silicon oil in eye	28	0.993	0.993	1.000	0.993	0.993	1.000	1.000	1.000	1.000	1.0000 (1.0000-1.0000)
Blur fundus	29	0.769	0.667	1.000	0.769	0.667	1.000	0.667	0.667	0.999	0.9419 (0.8296-1.0000)
**Referable, frequency-weighted average**		**0.955**	**0.943**	**0.998**	**0.960**	**0.950**	**0.998**	**0.961**	**0.968**	**0.998**	**0.9956**
**Subset accuracy, %**		**92.01**			**92.91**			**91.28**			

1. Zhang M, Zhou Z. A Review on Multi-Label Learning Algorithms. *IEEE Transactions on Knowledge and Data Engineering.* 2014;26(8):1819-1837.
2. Chilamkurthy S, Ghosh R, Tanamala S, et al. Deep learning algorithms for detection of critical findings in head CT scans: a retrospective study. *The Lancet.* 2018/12/01/ 2018;392(10162):2388-2396.
3. Ruder S. An Overview of Multi-Task Learning in Deep Neural Networks. *arXiv e-prints.* 2017. https://ui.adsabs.harvard.edu/abs/2017arXiv170605098R. Accessed June 01, 2017.
4. Boutell MR, Luo J, Shen X, Brown CM. Learning multi-label scene classification. *Pattern Recognition.* 2004/09/01/ 2004;37(9):1757-1771.
5. Team o_O solution for the Kaggle Diabetic Retinopathy Detection Challenge. 2015; https://github.com/sveitser/kaggle_diabetic.
6. Davis H, Russell SR, Barriga ES, Abr¨¤moff MD, Soliz P. Vision-based, real-time retinal image quality assessment. *2009 22nd IEEE International Symposium on Computer-Based Medical Systems.* 2009:1-6.
7. Graham* B. Kaggle Diabetic Retinopathy Detection Competition report 2015; https://www.kaggle.com/c/diabetic-retinopathy-detection/discussion/15801.
8. Orlando JI, Fu H, Barbosa Breda J, et al. REFUGE Challenge: A unified framework for evaluating automated methods for glaucoma assessment from fundus photographs. *Medical Image Analysis.* 2020/01/01/ 2020;59:101570.
9. Porwal P, Pachade S, Kokare M, et al. IDRiD: Diabetic Retinopathy – Segmentation and Grading Challenge. *Medical Image Analysis.* 2020/01/01/ 2020;59:101561.
10. Carmona E, rincon zamorano M, Martínez-de-la-Casa J. Identification of the optic nerve head with genetic algorithms. *Artificial intelligence in medicine.* 08/01 2008;43:243-259.
11. He K, Gkioxari G, Doll¨¢r P, Girshick R. Mask R-CNN. *ArXiv e-prints.* 2017;1703. http://adsabs.harvard.edu/abs/2017arXiv170306870H. Accessed March 1, 2017.
12. Zhou Z-H. Ensemble Learning. In: Li SZ, Jain AK, eds. *Encyclopedia of Biometrics*. Boston, MA: Springer US; 2015:411-416.
13. Szegedy C, Vanhoucke V, Ioffe S, Shlens J, Wojna Z. Rethinking the Inception Architecture for Computer Vision. *ArXiv e-prints.* 2015;1512. http://adsabs.harvard.edu/abs/2015arXiv151200567S. Accessed December 1, 2015.
14. Chollet Fo. Xception: Deep Learning with Depthwise Separable Convolutions. *ArXiv e-prints.* 2016;1610. http://adsabs.harvard.edu/abs/2016arXiv161002357C. Accessed October 1, 2016.
15. Szegedy C, Ioffe S, Vanhoucke V, Alemi A. Inception-v4, Inception-ResNet and the Impact of Residual Connections on Learning. *ArXiv e-prints.* 2016;1602. http://adsabs.harvard.edu/abs/2016arXiv160207261S. Accessed February 1, 2016.
16. Sandler M, Howard A, Zhu M, Zhmoginov A, Chen L-C. MobileNetV2: Inverted Residuals and Linear Bottlenecks. *ArXiv e-prints.* 2018;1801. http://adsabs.harvard.edu/abs/2018arXiv180104381S. Accessed January 1, 2018.
17. Tan M, Chen B, Pang R, Vasudevan V, Le QV. MnasNet: Platform-Aware Neural Architecture Search for Mobile. *ArXiv e-prints.* 2018;1807. http://adsabs.harvard.edu/abs/2018arXiv180711626T. Accessed July 1, 2018.
18. He K, Zhang X, Ren S, Sun J. Deep Residual Learning for Image Recognition. *ArXiv e-prints.* 2015;1512. http://adsabs.harvard.edu/abs/2015arXiv151203385H. Accessed December 1, 2015.
19. Xie S, Girshick R, Doll¨¢r P, Tu Z, He K. Aggregated Residual Transformations for Deep Neural Networks. *ArXiv e-prints.* 2016;1611. http://adsabs.harvard.edu/abs/2016arXiv161105431X. Accessed November 1, 2016.
20. He K, Zhang X, Ren S, Sun J. Identity Mappings in Deep Residual Networks. *ArXiv e-prints.* 2016;1603. http://adsabs.harvard.edu/abs/2016arXiv160305027H. Accessed March 1, 2016.
21. Huang G, Liu Z, van der Maaten L, Weinberger KQ. Densely Connected Convolutional Networks. *ArXiv e-prints.* 2016;1608. http://adsabs.harvard.edu/abs/2016arXiv160806993H. Accessed August 1, 2016.
22. A repository for Data Science Bowl 2017 competition.
23. Engstrom L, Tran B, Tsipras D, Schmidt L, Madry A. A Rotation and a Translation Suffice: Fooling CNNs with Simple Transformations. *arXiv e-prints.* 2017. https://ui.adsabs.harvard.edu/abs/2017arXiv171202779E. Accessed December 01, 2017.
24. Gwenole Quellec KC, Yassine Boudi, Mathieu Lamard. Deep image mining for diabetic retinopathy screening. *Medical Image Analysis.* 2017;39:178-193.
25. Image augmentation for machine learning experiments https://github.com/aleju/imgaug.
26. Cui Q, Yip HK, Zhao RC, So KF, Harvey AR. Intraocular elevation of cyclic AMP potentiates ciliary neurotrophic factor-induced regeneration of adult rat retinal ganglion cell axons. *Mol Cell Neurosci.* Jan 2003;22(1):49-61.
27. Müller R, Kornblith S, Hinton G. When Does Label Smoothing Help? *arXiv e-prints.* 2019. https://ui.adsabs.harvard.edu/abs/2019arXiv190602629M. Accessed June 01, 2019.
28. Tajbakhsh N, Shin JY, Gurudu SR, et al. Convolutional Neural Networks for Medical Image Analysis: Full Training or Fine Tuning? *IEEE Transactions on Medical Imaging.* 2016;35(5):1299-1312.
29. Yosinski J, Clune J, Bengio Y, Lipson H. How transferable are features in deep neural networks? *Proceedings of the 27th International Conference on Neural Information Processing Systems - Volume 2.* Montreal, Canada: MIT Press; 2014:3320-3328.
30. Kingma DP, Ba J. Adam: A Method for Stochastic Optimization. *ArXiv e-prints.* 2014;1412. http://adsabs.harvard.edu/abs/2014arXiv1412.6980K. Accessed December 1, 2014.

31. Zhang MR, Lucas J, Hinton G, Ba J. Lookahead Optimizer: k steps forward, 1 step back. *arXiv e-prints.* 2019. https://ui.adsabs.harvard.edu/abs/2019arXiv190708610Z. Accessed July 01, 2019.
32. Guo C, Pleiss G, Sun Y, Weinberger KQ. On calibration of modern neural networks. *Proceedings of the 34th International Conference on Machine Learning - Volume 70.* Sydney, NSW, Australia: JMLR.org; 2017:1321-1330.
33. Ju C, Bibaut Al, van der Laan MJ. The Relative Performance of Ensemble Methods with Deep Convolutional Neural Networks for Image Classification. *arXiv e-prints.* 2017. https://ui.adsabs.harvard.edu/\#abs/2017arXiv170401664J. Accessed April 01, 2017.
34. Amirata Ghorbani AA. Interpretation of Neural Networks Is Fragile. *AAAI 2019*2019.
35. Zhou B, Khosla A, Lapedriza A, Oliva A, Torralba A. Learning Deep Features for Discriminative Localization. *ArXiv e-prints.* 2015;1512. http://adsabs.harvard.edu/abs/2015arXiv151204150Z. Accessed December 1, 2015.
36. Lundberg S, Lee S-I. A Unified Approach to Interpreting Model Predictions. *arXiv e-prints.* 2017. https://ui.adsabs.harvard.edu/\#abs/2017arXiv170507874L. Accessed May 01, 2017.
37. Shrikumar A, Greenside P, Kundaje A. Learning Important Features Through Propagating Activation Differences. *arXiv e-prints.* 2017. https://ui.adsabs.harvard.edu/\#abs/2017arXiv170402685S. Accessed April 01, 2017.
38. Sundararajan M, Taly A, Yan Q. Axiomatic Attribution for Deep Networks. *arXiv e-prints.* 2017. https://ui.adsabs.harvard.edu/\#abs/2017arXiv170301365S. Accessed March 01, 2017.
39. Chattopadhyay A, Sarkar A, Howlader P, Balasubramanian VN. Grad-CAM++: Generalized Gradient-based Visual Explanations for Deep Convolutional Networks. *ArXiv e-prints.* 2017;1710. http://adsabs.harvard.edu/abs/2017arXiv171011063C. Accessed October 1, 2017.
40. TensorFlow. https://www.tensorflow.org/.
