# Supplementary material for: Automatic detection of 39 fundus diseases and conditions in retinal photographs using deep neural networks
Source: Nat Commun. 2021 Aug 10;12:4828. doi: 10.1038/s41467-021-25138-w (PMC8355164; doi:10.1038/s41467-021-25138-w)
Supplement: Supplementary file 3 — Reporting Summary [file 41467_2021_25138_MOESM3_ESM.pdf]

nature portfolio

Corresponding author(s): Mingzhi Zhang

Last updated by author(s): Jun 14, 2021

Reporting Summary

Nature Portfolio wishes to improve the reproducibility of the work that we publish. This form provides structure for consistency and transparency in reporting. For further information on Nature Portfolio policies, see our Editorial Policies and the Editorial Policy Checklist.

Statistics

For all statistical analyses, confirm that the following items are present in the figure legend, table legend, main text, or Methods section.

n/a	Confirmed	
☐	☒	The exact sample size (n) for each experimental group/condition, given as a discrete number and unit of measurement
☐	☒	A statement on whether measurements were taken from distinct samples or whether the same sample was measured repeatedly
☒	☐	The statistical test(s) used AND whether they are one- or two-sided<br>*Only common tests should be described solely by name; describe more complex techniques in the Methods section.*
☒	☐	A description of all covariates tested
☒	☐	A description of any assumptions or corrections, such as tests of normality and adjustment for multiple comparisons
☐	☒	A full description of the statistical parameters including central tendency (e.g. means) or other basic estimates (e.g. regression coefficient) AND variation (e.g. standard deviation) or associated estimates of uncertainty (e.g. confidence intervals)
☒	☐	For null hypothesis testing, the test statistic (e.g. F, t, r) with confidence intervals, effect sizes, degrees of freedom and P value noted<br>*Give P values as exact values whenever suitable.*
☒	☐	For Bayesian analysis, information on the choice of priors and Markov chain Monte Carlo settings
☐	☒	For hierarchical and complex designs, identification of the appropriate level for tests and full reporting of outcomes
☒	☐	Estimates of effect sizes (e.g. Cohen's d, Pearson's r), indicating how they were calculated

Our web collection on statistics for biologists contains articles on many of the points above.

Software and code

Policy information about availability of computer code

Data collection	Data collection was performed with custom software written in Python (3.5) and the following software packages: OpenCV (3.4), pandas (0.24.1), MySQL(5.7.23), MySQLClient (1.4.6), Numpy (1.15.4) .
Data analysis	Analysis was performed with custom code written in Python (3.5), and the following software packages were used: Tensorflow (1.12), Keras (2.2.4), OpenCV (3.4), shap (0.26), Numpy (1.15.4), Django (2.1.7), CUDA (9.2), cuDNN (7.2.1), pandas (0.24.1), scikit-learn (0.20.2) and a R (3.6.3) package pROC (1.14.0). Code for image preprocessing is available on Github (https://github.com/linchundan88/Fundus-image-preprocessing). Code for training, validation, test and RPC service is available on Github (https://github.com/linchundan88/fundus_multiple_diseases). Code for web application is available on Github (https://github.com/linchundan88/fundus_multiple_diseases_web)

For manuscripts utilizing custom algorithms or software that are central to the research but not yet described in published literature, software must be made available to editors and reviewers. We strongly encourage code deposition in a community repository (e.g. GitHub). See the Nature Portfolio guidelines for submitting code & software for further information.

Data

Policy information about availability of data

All manuscripts must include a data availability statement. This statement should provide the following information, where applicable:

- Accession codes, unique identifiers, or web links for publicly available datasets
- A description of any restrictions on data availability
- For clinical datasets or third party data, please ensure that the statement adheres to our policy

Dataset form EYEPACS Kaggle for training, validation and test is available at (https://www.kaggle.com/c/diabetic-retinopathy-detection). Public datasets for test are

available at (Messidor-2: https://www.adcis.net/en/third-party/messidor2/, IDRID: https://idrid.grand-challenge.org/, REFUGE: https://refuge.grand-challenge.org/, PALM: https://palm.grand-challenge.org/). Other datasets supporting the findings of the current study are not publicly available due to the confidential policy of National Health Commission of China, but are available from the corresponding author upon reasonable request.

Field-specific reporting

Please select the one below that is the best fit for your research. If you are not sure, read the appropriate sections before making your selection.

☒ Life sciences ☐ Behavioural & social sciences ☐ Ecological, evolutionary & environmental sciences

For a reference copy of the document with all sections, see nature.com/documents/nr-reporting-summary-flat.pdf

Life sciences study design

All studies must disclose on these points even when the disclosure is negative.

Sample size	In total, 249,620 images marked with 275,543 labels were collected for algorithm training, validation and tests. The training dataset of 129,264 images was collected from JSIEC (n= 74,683), LEDRS (n= 27,463) and EyePACS (n= 27,118). The validation and tests datasets contained 120,356 images that had not been “seen” by the algorithm during training process. They consisted of five parts. 1) Primary validation dataset (n= 22,800) collected from JSIEC (n= 13,247), LEDRS (n= 4,787) and EyePACS (n= 4,766). 2) Primary test dataset (n= 27,611 collected from JSIEC 2018 (n= 14,502), LEDRS 2018 (n= 7,052) and EyePACS (n= 6,057). 3) External multihospital test datasets (n= 60,445) collected from three hospitals, Fujian (n= 39,671), Tibet (n= 14,826) and Xinjiang (n= 5,948). 4) Tele-reading application dataset (n= 6,062) from 7 primary hospitals in different part of China, 5) The public test dataset (n=3,438) was collected from three publicly available datasets: Messidor-2 (n=1,748), IDRID (n=516), PALM) (n= 374), and REFUGE (n=800). The total sample size for training, validation and test sets was informed by the existing literature and in the field of deep learning on fundus retinal images (Gulshan et al., 2016; Ting et al., 2017). Large amounts of data are required to determine the millions of parameters during training of the deep neural networks.
Data exclusions	Images collected from various data sources were included according to pre-established criteria of quality and description of diseases and conditions. Firstly, images were screened by an automatic quality control algorithm, scoring lower than 80 (0-100) were discarded. Secondly, unclassifiable images were also excluded, which were firstly judged by unspecialized ophthalmologists and senior retina specialists and further confirmed by the retina experts.
Replication	All attempts at replication were successful. Our findings persisted through numerous retrainings with random network initialization and training data iteration order. The high performance of our models was replicated on the completely independent internal, external, and public datasets.
Randomization	Code for partition was run with random seeds for reproducibility. Image data from JSIEC and LEDRS was firstly divided into two parts according the collecting date. The part before 2018 was randomly split into a training set (85%) and validation set (15%) on a per-case basis. The other part collected within 2018 was applied as test dataset. Same randomized partition in case based for all images from EyePACS was conducted without collection date information.
Blinding	Participants in the clinical evaluation and image labeling for the DLP training, validation and testing were blinded to the ground truth and were not involved in dataset collection. Patients who had previously been treated in clinic by the participants were excluded from the comparative set.

Reporting for specific materials, systems and methods

We require information from authors about some types of materials, experimental systems and methods used in many studies. Here, indicate whether each material, system or method listed is relevant to your study. If you are not sure if a list item applies to your research, read the appropriate section before selecting a response.

Materials & experimental systems

n/a	Involved in the study	
☒	☐	Antibodies
☒	☐	Eukaryotic cell lines
☒	☐	Palaeontology and archaeology
☒	☐	Animals and other organisms
☐	☐	Human research participants
☒	☐	Clinical data
☒	☐	Dual use research of concern

Methods

n/a	Involved in the study	
☒	☐	ChIP-seq
☒	☐	Flow cytometry
☒	☐	MRI-based neuroimaging

Human research participants

Policy information about studies involving human research participants

Population characteristics	The dataset of JSIEC was collected from the PACS between Sep 2009 and Dec 2018, and the images were taken by a ZEISS

Population characteristics	FF450 Plus IR Fundus Camera (2009-2013) and Topcon TRC-50DX Mydriatic Retinal Camera (2013-2018) in a 35-50°field setting. The LEDRS dataset was obtained from thirteen hospitals located at different parts of China between April 2014 and Dec 2018. The tele-reading dataset (n=6,062) for application was collected from seven primary hospitals or community health centers in different parts of China. The EyePACS images were macular-centered fundus images obtained from the EyePACS public dataset (EyePACS LLC, Berkeley, CA), which is a telemedicine program for diabetic retinopathy screening in community clinics across the United States. The Messidor-2 dataset was obtained from 4 French eye institutions acquired with a Topcon TRC NW6 nonmydriatic camera. The IDRID dataset was established for "Diabetic Retinopathy: Segmentation and Grading Challenge" workshop at IEEE International Symposium on Biomedical Imaging (ISBI-2018). E-ophtha is a database of color fundus images especially designed for scientific research in Diabetic Retinopathy (DR). It has been generated from the OPHDIAT© Tele-medical network for DR screening, in the framework of the ANR-TECSAN-TELEOPHTA project funded by the French Research Agency (ANR).<br>The primary datasets for training, validation and test consisted of 179,675 images from 96,900 subjects (mean age, 51.6 years, 49.9% men in the JSIEC set; mean age, 62.2 years, 43.5% men in the LEDRS set; prevalence of referable, 68.7%). The external multihospital test sets had 60,445 images from 29,343 subjects (mean age, 55.4 years, 50.3%men in Tibet set; prevalence of referable, 66.2%). Approximately 97.1% were obtained from subjects of Tibetan ethnicity in the Tibet dataset and about 13.3% of the images were acquired from subjects of Uyghur ethnicity in Sinkiang dataset. The tele-reading categorized dataset had 6,062 images from 3,251 subjects.
Recruitment	Image data of the JSIEC dataset were collected from patients having clinical care in JSIEC between Sep 2009 and Dec 2018, who had fundus photography and met the inclusion criteria. The images were taken by a ZEISS FF450 Plus IRFundus Camera (2009-2013) and Topcon TRC-50DXMydriatic Retinal Camera (2013-2018) in a 35-50°field setting. Data of LEDRS were collected from patients attending the LEDRS project in thirteen hospitals located at different parts of China (Guilin City Second Hospital, Jilin City Chinese Traditional and Western Medicine Hospital, Jinan City Lixia District People's Hospital, Luoyang City Third Hospital, Luzhou City Red Cross Hospital, Huhehaote Neimengu Province People Hospital, Zhanjiang City Second Hospital, Zhengzhou City Second Hospital, Beihai People's Hospital, Zhoukou City Eye Hospital, Nanyang City Ninth People's Hospital, Chongqing Wanzhou District People's Hospital, Liuzhou Red Cross Hospital) between April 2014 and Dec 2018, who had fundus photography met the inclusion criteria. Data of the tele-reading application set were collected from hospitals or centers belong to the JSIEC-Specialized Treatment Combination (JSTC) between July 2019 and May 2020, which includes Balinzuoqi Hospital of Traditional Mongolian Medicine and Chinese Medicine (Inner Mongolia), Hainan Tibetan Autonomous Prefecture People's Hospital (Qinghai), Nyingchi People's Hospital (Tibet), DuShanzi People's Hospital (Xinjiang), Sanrao Community Health center (Guangdong), Huizhai Community Health center (Guangdong), Zhongshan Torch Development Zone Hospital (Guangdong). For more details please refer to the manuscript methods and results sections.
Ethics oversight	Human Ethics Committee of JSIEC

Note that full information on the approval of the study protocol must also be provided in the manuscript.
